# Antitumor Activity and Multi-Target Mechanism of Phenolic Schiff Bases Bearing Methanesulfonamide Fragments: Cell Cycle Analysis and a Molecular Modeling Study

**DOI:** 10.3390/ijms252413621

**Published:** 2024-12-19

**Authors:** Alaa A.-M. Abdel-Aziz, Adel S. El-Azab, Simone Brogi, Rezk R. Ayyad, Ibrahim A. Al-Suwaidan, Mohamed Hefnawy

**Affiliations:** 1 Department of Pharmaceutical Chemistry, College of Pharmacy, King Saud University, P.O. Box 2457, Riyadh 11451, Saudi Arabia; adelazab@ksu.edu.sa (A.S.E.-A.); ialsuwidan@ksu.edu.sa (I.A.A.-S.); mhefnawy@ksu.edu.sa (M.H.); 2 Department of Pharmacy, University of Pisa, Via Bonanno 6, 56126 Pisa, Italy; simone.brogi@unipi.it; 3 Department of Pharmaceutical Chemistry, College of Pharmacy, University of Hilla, Babylon 6202, Iraq; rezkayyad.222@azhar.edu.eg

**Keywords:** phenolic Schiff bases, antitumor activity, enzymatic assay, cell cycle analysis, molecular docking, molecular dynamic simulation

## Abstract

Five phenolic Schiff bases (**7**–**11**) incorporating a fragment of methanesulfonamide were synthesized and evaluated for their efficacy as antitumor agents. Compounds **7** and **8** demonstrated the most potent antitumor action, with a positive cytotoxic effect (PCE) of 54/59 and 59/59 and a mean growth percentage (MG%) of 67.3% and 19.5%, respectively, compared with imatinib (PCE = 20/59 and MG% = 92.6%). The PCE values for derivatives **9**–**11** were 3/59, 4/59, and 4/59, respectively, indicating poor antitumor effect. Compound **8** exhibited the most significant efficacy, suppressing cell proliferation by an average of 50% at a dosage of 0.501 µM, in comparison with the reference drugs sorafenib (2.33 µM), gefitinib (2.10 µM), erlotinib (7.68 µM), and celecoxib (17.5 µM). Compounds **7** and **8** had substantial inhibitory effects on the human epidermal growth factor receptor 2 (HER2), with IC_50_ values of 0.183 μM and 0.464 μM, respectively. Furthermore, they exhibited significant inhibition of the epidermal growth factor receptor (EGFR), with IC_50_ values of 0.752 μM and 0.166 μM, respectively. Compound **8** exhibited the highest COX-2 inhibition (IC_50_ = 12.76 μM). We performed molecular docking dynamic experiments to examine the precise interaction and structural prerequisites for the anticancer activity of derivatives **7** and **8** by targeting EGFR and HER2.

## 1. Introduction

Cancer is a major cause of death worldwide [1,2]. Furthermore, cancer cells can acquire resistance to the existent chemotherapeutic agents [3]. Accordingly, there is a significant demand for novel and effective compounds that can fight against tumors [4,5,6,7,8,9,10,11,12,13,14,15,16,17,18,19,20,21,22,23]. Developing such substances presents a challenge in drug discovery because of the adverse effects associated with the combination of multiple medications used in cancer therapy [3,24]. The recommended therapeutic approach to reduce these consequences is to utilize a single drug that can engage in several molecular pathways, decreasing their impact [25,26,27,28,29,30,31,32,33,34,35].

**Figure 1:**
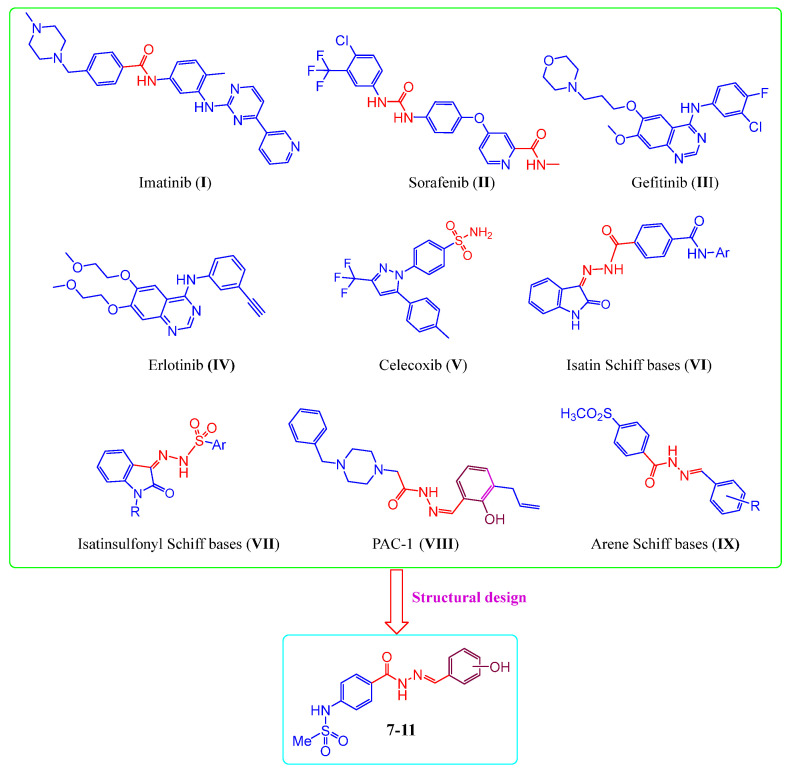
The reported anticancer agents and designed phenolic Schiff bases **7**–**11**.

HER2 and EGFR possess structural similarities and are part of the tyrosine kinase receptor family [36,37,38,39,40,41,42,43]. HER2 belongs to the epidermal growth factor receptor, which has tyrosine kinase activity [37,38,39,40]. The receptor dimerization leads to the autophosphorylation of tyrosine residues within the receptor’s cytoplasmic domain. This result promotes different signaling pathways, causing tumorigenesis and cell proliferation [37,38,39,40]. HER2 overexpression occurs in approximately 15–30% of breast cancers. It has also been detected in other cancers, such as the ovary, colon, lung, and bladder [37,38,39,40]. In addition, EGFR plays a crucial role in cancer. It regulates the cell signaling pathways that control cell division and survival. Mutations in the EGFR gene result in its overexpression, leading to tumor growth, invasion, and metastasis [41,42,43]. The overexpression of HER2 and EGFR has been observed in several malignancies, such as breast, prostate, colon, and ovarian cancers [36,37,38,39,40,41,42,43]. It has been demonstrated that blocking these proteins can trigger programmed cell death in breast and lung malignancies [37,38,39,40,41,42,43]. Tyrosine kinase inhibitors are used to treat various types of cancer [38,44,45,46,47,48]. Examples of these drugs include imatinib (**I**), sorafenib (**II**), gefitinib (**III**), and erlotinib (**IV**) (Figure 1) [49,50,51,52]. In addition, the COX-2 isozyme is excessively produced in several malignancies, such as gastric, lung, colon, breast, ovarian, prostate, and hepatocellular cancers [53]. These findings indicate that COX-2 can be explicitly used to treat cancer [54]. Celecoxib (**V**), a selective COX-2 inhibitor (Figure 1), has the potential to be used in the treatment and prevention of cancer [55]. The hypothesized mechanism by which COX-2 inhibition may fight cancer involves reducing cell growth or inducing apoptosis [56].

Schiff’s bases are a crucial and biologically potent pharmacophore that aids in developing several anticancer drugs [48,57]. Examples of these compounds include isatin Schiff bases (**VI** and **VII**) and PAC-1 (**VIII**), some of which initiate programmed cell death (apoptosis) in cancer cells (Figure 1) [58,59,60]. Moreover, compounds incorporating methylsulfonyl fragments, such as arene Schiff bases (**IX**), can function as antitumor agents against a range of cancers, including skin, hepatic, colon, and lung cancers, as well as melanoma (Figure 1) [61]. Moreover, the potential of several Schiff bases to selectively inhibit EGFR, HER2, and COX-2, as well as cause apoptosis, has been investigated concerning their anticancer properties [57,58,59,60,61,62]. Moreover, other Schiff bases can hinder the activity of the COX-2 enzyme and tumor-associated carbonic anhydrase [62,63,64,65,66,67].

In our investigation, we synthesized five phenolic Schiff’s bases (compounds **7**–**11**) containing a methane sulfonamide moiety (Figure 1). The structure of the created compounds was formed from an azomethine fragment, which is the link between the methanesulfonamide core and the phenolic moiety. The anticancer efficacy of the compounds was evaluated in 59 human cancer cell lines via in vitro testing. Afterward, a cell cycle analysis and an experiment to induce apoptosis were performed using the most effective derivative in MCF-7 cancer cells. We conducted an enzymatic experiment to determine the strongest inhibitory effects of the derivatives on EGFR/HER2 and COX-2 enzymes. Finally, we used a molecular modeling tool to investigate how the biologically active molecules can interact with EGFR and HER2 tyrosine kinase binding sites.

## 2. Results and Discussion

### 2.1. Chemistry

**Scheme 1:**
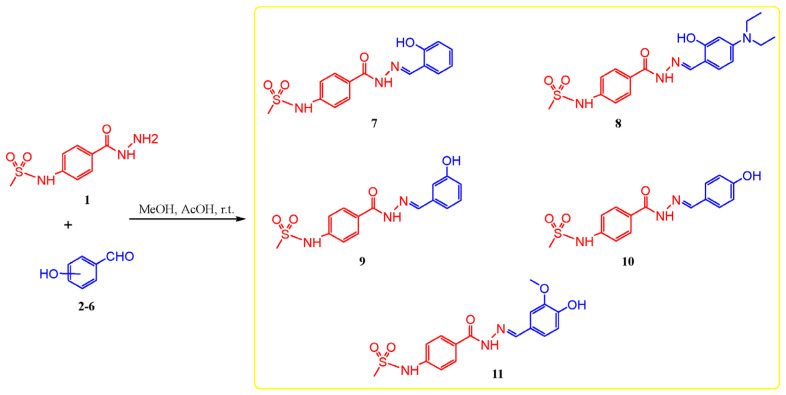
Synthesis of the designed phenolic Schiff bases **7**–**11**.

According to our previous study, (E)-N-(4-(2-arylidenehydrazine-1-carbonyl)phenyl)methanesulfonamides **7**–**11** were synthesized with a reasonable yield by mechanically mixing a phenolic aldehyde and N-(4-(hydrazine carbonyl)phenyl)methanesulfonamide (**1**) in methanol as a solvent and acetic acid as a catalyst at room temperature (Scheme 1). NMR and other spectra were used to verify the structures of the target derivatives, as described in our previous report [66].

### 2.2. Antitumor Activity Results

#### 2.2.1. Growth Inhibition Percentage (GI%) at a Single Dose Concentration of 10 µM

The National Cancer Institute (Bethesda, MD, USA) selected compounds **7**–**11** for the screening of antitumor efficacy against a comprehensive panel of 59 cancer cell lines derived from nine human tissues (i.e., brain, blood, colon, skin, lung, kidney, ovary, breast, and prostate) [19,61,68]. Table 1 presents the preliminary antitumor assessment at a concentration of 10 µM and growth inhibition percentage (GI%) across 59 cell lines compared with imatinib as the reference drug (Table 1).

Compounds **7**–**11** demonstrated noteworthy antitumor efficacy, with a mean growth percentage (MG) of 101.84%–19.55% and a PCE (ratio between the number of cell lines with percentage growth inhibition from ≥10 to 100 and the total number of cell lines) of 3/59–59/59.

Phenolic Schiff bases **7** and **8** exhibited the highest PCE of 54/59 and 59/59 (MG = 67.3% and 19.5%, respectively), while compounds **9**–**11** possessed the lowest PCE of 4/59 (MG = 101.8%–97.6%). Imatinib had PCE = 20/55 and MG = 92.6%. Interestingly, 2-phenolic Schiff bases 7 and 8 exhibited more potent antitumor effects (PCE = 54/59 and 59/59) than their 3-phenolic and 4-phenolic counterparts, such as derivatives 9, 10, and 11 (PCE = 3/59, 4/59, and 4/59, respectively). Schiff bases containing the N-diethylamino moiety, like compound **8** (MG = 19.5%), were more effective antitumor agents than compound **7** (MG = 67.3%). The broad-spectrum efficacy and selectivity of Schiff bases **7**–**11** (Table 1) against cancer tissues (59 cell lines) were compared with imatinib (GI ≥ 10%–47.1%). It was clear that derivatives **7** and **8** had potent GI (>10%–100%) against the majority of the cell lines tested, including melanoma (GI = 22.0%–100.0%), leukemia (GI = 22.0%–99.0%), non-small cell lung (GI = 11.0%–97.0%), colon (GI = 35.0%–94.0%), renal (GI = 16.0%–100.0%), CNS (GI = 32.0%–93.0%), prostate (GI = 33.0%–69.0%), ovarian (GI = 23.0%–88.0%), and breast (GI = 11.0%–100.0%). Weak to moderate antitumor efficacy was demonstrated by derivatives **9**, **10**, and **11** against leukemia (GI = 10.0%), NSCLC (GI = 23.0%), melanoma (GI = 10.0%–12.0%), renal cancer (GI = 10.0%–17.0%), and breast cancer (GI = 11.0%). Imatinib, on the other hand, had a moderate effect on most types of cancer cells, including leukemia (GI = 12.6%–18.0%), NSCLC (GI ≥ 10.0%–17.1%), colon (GI = 11.5% to 47.1%), CNS (GI ≥ 10.0%–24.5%), melanoma (GI = 11.6%–22.3%), ovarian (GI ≥ 10.0%), renal (GI ≥ 10.0%–13.7%), prostate (GI ≥ 10.0%–14.4%), and breast (GI = 11.2%–29.1%).

#### 2.2.2. GI_50_, TGI, and LC_50_ of Compound **8** and Comparison with Reference Drugs

**Figure 2:**
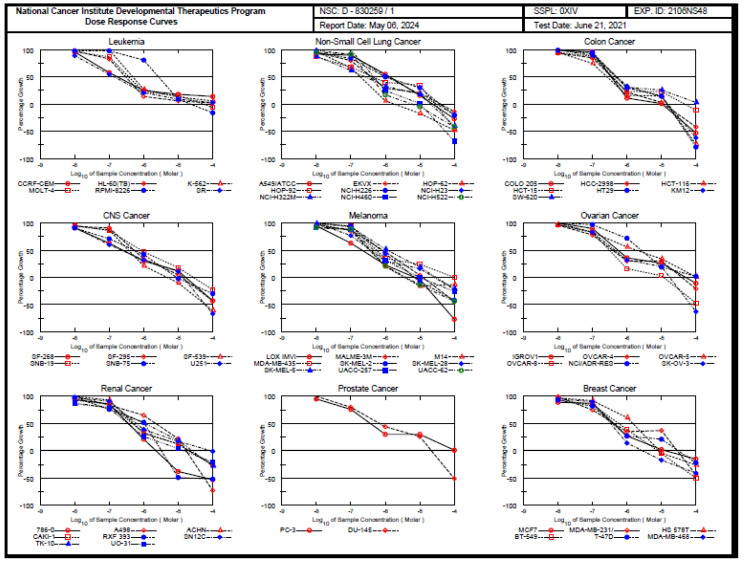
Dose−response curve of compound **8**.

The most potent broad-spectrum antitumor agent was Schiff base 8. The antitumor effects were observed at five different concentrations (ten-fold dilution, 100, 10, 1, 0.1, and 0.01 µM) using 59 different types of cancer cells (Figure 2 and Table 2 and Table 3). The potencies of sorafenib, gefitinib, erlotinib, and celecoxib as reference medications were compared with the assay results (Table 2 and Table 3). In Table 3, we determined three dose–response measures of the antitumor effect of compound **8** and the common antitumor agents for every cell line: median growth inhibition (GI_50_), total growth inhibition (TGI), and median lethal concentration (LC_50_) [19,61,68]. Furthermore, an average activity parameter for phenolic Schiff base **8** and reference drugs was derived across all the cell lines by computing the mean GI_50_ graph midpoints (GI_50_ MG_MID) for these parameters. With a GI_50_ range of 0.141–2.72 µM, derivative **8** exhibited potent antitumor potency, as shown in Table 2 and Table 3. These values were compared with those of sorafenib, gefitinib, erlotinib, and celecoxib, with GI_50_ values ranging from 1.26 to 3.98 µM, 0.01258 to 10.0 µM, 0.10 to 100.0 µM, and 3.98 to 63.09 µM, respectively (Table 3).

Phenolic imine **8** exhibited notable antitumor efficacy in multiple human cancers, with the mean GI_50_ values recorded as follows: 0.70 µM for leukemia, 0.622 µM for NSCLC, 0.40 µM for colon cancer, 0.451 µM for CNS cancer, 0.563 µM for melanoma, 1.027 µM for ovarian cancer, 0.826 µM for renal cancer, 0.527 µM for prostate cancer, and 0.599 µM for breast cancer (Table 2 and Table 3).

The mean GI_50_ values for sorafenib, gefitinib, erlotinib, and celecoxib are shown in Table 3: CNS cancer (2.33, 5.64, 16.99, and 17.22 µM, respectively); NSCLC (2.34, 2.05, 13.59, and 25.09 µM, respectively); melanoma (1.87, 3.68, 23.74, and 22.46 µM, respectively); colon cancer (2.19, 5.23, 51.68, and 20.73 µM, respectively); ovarian cancer (2.89, 3.05, 5.52, and 17.14 µM, respectively); renal cancer (2.86, 1.41, 2.46, and 19.87 µM, respectively); prostate cancer (2.58, 3.29, 20.90, and 14.22, µM, respectively); and breast cancer (2.17, 4.67, 24.72, and 17.49 µM, respectively).

A comparative analysis of the antitumor effect of **8** against standard drugs revealed that the potency compound **8** (GI_50_ MG_MID = 0.501 µM) was approximately four-fold higher than sorafenib (GI_50_ MG_MID = 2.33 µM), three-fold higher than gefitinib (GI_50_ MG_MID = 2.1µM), eleven-fold higher than erlotinib (GI_50_ MG_MID = 7.68 µM), and thirteen-fold higher than celecoxib (GI_50_ MG_MID = 17.5µM).

### 2.3. In Vitro Cytotoxicity Against Normal Human Cell

To examine the therapeutic safety of the newly synthesized hybrids and assess their selective cytotoxicity toward normal and tumor cells, a standard fibroblast-like fetal lung cell line (WI−38) was used. In contrast, standard anticancer drugs, such as lapatinib and staurosporine, were used. Compared with lapatinib and staurosporine, which had IC_50_ values of 32.01 and 19.76 μM on normal cells, derivatives **7** and **8** showed less cytotoxicity against WI−38 normal fibroblast cells, with IC_50_ values of 110.55 and 82.12 μM, respectively.

### 2.4. Enzymatic Inhibition Assay

#### EGFR/HER2 Kinase and COX−2 Inhibition Activities [47,61,69]

The most potent Schiff bases **7** and **8** were evaluated to confirm their inhibitory potency against the EGFR/HER2 kinase and COX−2 enzyme. The inhibitory effects of **7**, **20**, and the standard drugs erlotinib and lapatinib are presented in Table 4. The results are expressed as IC_50_ (μM) as the mean of three independent experiments. The IC_50_ of erlotinib and lapatinib against EGFR were 0.105 and 0.045 μM, respectively. The IC_50_ values using HER2 were 0.085 and 0.033 μM, respectively. The IC_50_ values of derivatives **7** and **8** against EGFR were 0.752 and 0.166 μM, respectively, and HER2 were 0.283 and 0.464 µM, respectively. Derivative **8** had an inhibitory effect similar to that of Erlotinib against EGFR.

On the other hand, the COX–2 inhibition assay was performed using compounds **7**, **8**, celecoxib, and ibuprofen, as shown in Table 4. The IC_50_ values of celecoxib and ibuprofen were 2.90 and 3.065 μM, respectively. Compound **7** exhibited significantly low COX-2 inhibition (IC_50_ = 20.86), whereas derivative **8** demonstrated moderate COX-2 inhibition (IC_50_ = 12.76 μM).

### 2.5. Annexin V–FITC Apoptosis Assay and Cell Cycle Analysis

**Figure 3:**
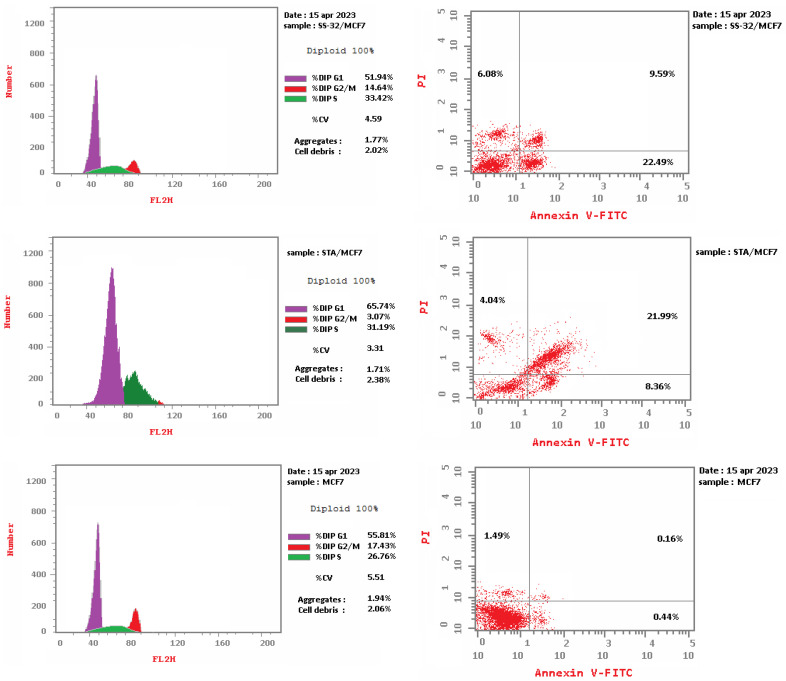
Effects of compound **8** (**upper right panel**), Staurosporine (**middle right panel**), and DMSO (**lower right panel**) on the percentage of annexin V-FITC-positive staining in MCF-7 cells and cell cycle analysis of MCF-7 cells treated with compound **8** (**upper left panel**), Staurosporine (**middle left panel**), and DMSO (**lower left panel**).

Apoptosis is the primary mechanism by which potent chemotherapeutic agents destroy cancer cells [70,71]. To distinguish between the apoptosis and the necrosis mode of MCF-7 cell death caused by the most active compound **8** and reference drug Staurosporine, we performed a cytometric analysis using the BD FACSCalibur (BD Biosciences, San Jose, CA, USA) annexin V-fluorescein isothiocyanate (FITC)/propidium iodide (AV/PI) dual-staining assay (Table 5) [71]. We stained MCF-7 cells with AV/PI for 24 h at a mixed molar concentration of 10 M using compound **8**. The effects of treating MCF-7 cells with compound **8** and Staurosporine for 24 h are presented in Figure 3 and Table 5. The early apoptosis ratio in Figure 3 rises sharply from 0.44% in the control sample (DMSO) to 22.49% in the lower-right quadrant of the cytogram of compound **8** and 8.36% for Staurosporine. Similarly, the upper-right quadrant of the cytogram shows an increase in the late apoptosis ratio from 0.16% in the control sample to 9.59% and 21.99% in derivative **8** and Staurosporine, respectively. Rather than supporting the necrotic pathway, these data support the apoptotic mechanism underlying the programmed cell death induced by compound **8** and Staurosporine. Flow cytometry measured cell growth in the G0-G1, S, and G2/M cell cycle phases [72]. We selected compound **8**, the most active molecule in the series, to further investigate its effects on the cell cycle progression of the MCF-7 cell line (Figure 3 and Table 5). Staurosporine was used as a standard drug, and DMSO was a negative control. Compound **8** was incubated in MCF-7 cells for 24 h at a concentration of 10 μM. Compound **8** interfered with the normal cell cycle of the MCF-7 cells. An increase in cells in the S phase (33.42%) compared with control (26.76%) suggests a significant influence on the percentage of apoptotic cells, leading to cell cycle arrest. The rate of cells in the G2/M phase decreased significantly from 17.43% in the control group to 14.64% (Figure 3 and Table 5). Staurosporine exhibited an increase in the S phase (31.19%) and G1 phase (65.74%), leading to cell cycle arrest (Figure 3 and Table 5).

### 2.6. Apoptotic Regulators (Bax, Bcl−2, Caspase−8, and Caspase−9)

**Figure 4:**
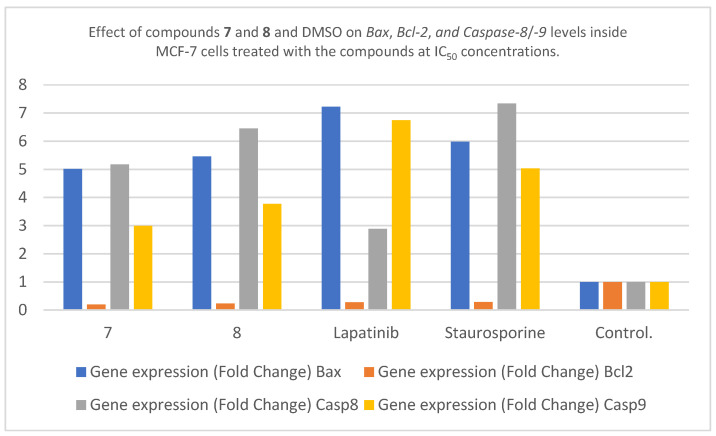
Effect of compound **8,** lapatinib, and staurosporine on *Bax*, *Bcl*−*2*, and *Caspase*−*8/*−*9* levels inside MCF−7 cells.

This study used MCF−7 cells and derivatives **7** and **8** at their IC_50_ concentrations. We measured the apoptosis promoter *Bax* levels, the apoptosis inhibitor *Bcl*−2, and the apoptosis coordination enzymes *caspase-8* and *caspase-9* [32]. Table 6 and Figure 4 illustrate that the MCF−7 cells treated with compounds **7** and **8** expressed more *Bax* protein than the untreated control cells, with fold changes of 5.02 and 5.456, respectively, compared with the untreated control cells. The standard drugs lapatinib and staurosporine exhibited fold changes of 7.224 and 5.987, respectively. The treatment of compounds **7** and **8** with MCF−7 significantly reduced the expression of the apoptosis inhibitor *Bcl-2* protein, with fold changes of 0.201 and 0.232, respectively, compared with lapatinib and staurosporine, which showed fold changes of 0.278 and 0.281, respectively. Furthermore, the levels of *caspase*−*8* and *caspase*−*9* were effectively increased when treating MCF−7 cells with derivatives **7** (fold changes of 5.171 and 2.99, respectively) and **8** (fold changes of 6.456 and 3.776, respectively) compared with the untreated control. Additionally, these levels increased when using the reference drugs lapatinib (fold changes of 2.887 and 6.745, respectively) and staurosporine (fold changes of 4.342 and 5.034, respectively).

### 2.7. Molecular Modeling Study

Molecular modeling is a valuable technique for investigating the binding mode of ligand molecules within receptor or enzyme-binding sites and the activity and SAR of bioactive compounds [73].

#### 2.7.1. Molecular Docking Analysis

**Figure 5:**
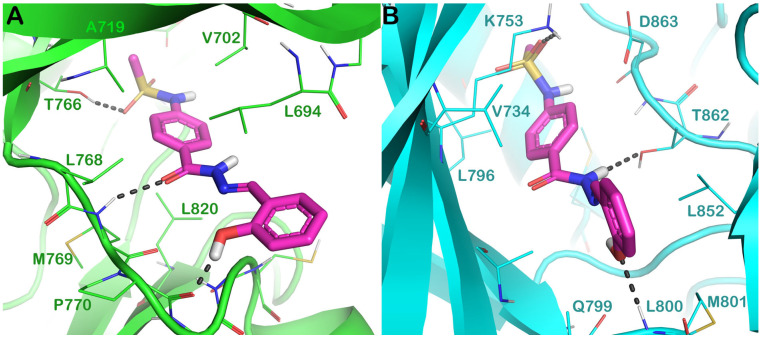
Docked poses of compound **7** (magenta sticks) within the EGFR (panel (**A**), PDB ID 1M17) and HER2 (panel (**B**), PDB ID 3RCD) binding sites. The interacting residues at the binding sites are indicated by lines and are labeled. The black dotted lines represent H-bonds. The pictures were generated by PyMOL (the PyMOL Molecular Graphics System, v1.8; Schrödinger, LLC, New York, NY, USA 2015).

To gain insight into the mechanism of action of the selected compounds, we conducted computational studies to investigate the potential binding modes of the most potent compounds in inhibiting EGFR and HER2 proteins. In particular, by combining molecular docking with molecular dynamics simulation, we proposed potential binding modes governing compounds **7** and **8**’s strong inhibitory activity against these enzymes. Figure 5 shows the molecular docking results for compound **7**. The in silico results showed that derivative **7** can strongly interact within the EGFR binding site via a series of polar contacts and hydrophobic interactions (Figure 5A). The compound established H-bonds with the sidechain of T766 and the backbones of M769 and P770. In addition, hydrophobic interactions with L694, V702, L768, and L820 were also detected. This docking pose accounted for a docking score of −7.834 kcal/mol, indicating the remarkable affinity of this compound for the selected binding site.

For HER2, we observed a series of H-bonds with K753, T862, and L800. Furthermore, hydrophobic interactions with residues V734, L796, L800, M801, and L852 were observed (Figure 5B). A docking score of −7.648 kcal/mol indicated significant binding affinity for the enzyme, as determined by the experimental evaluation.

**Figure 6:**
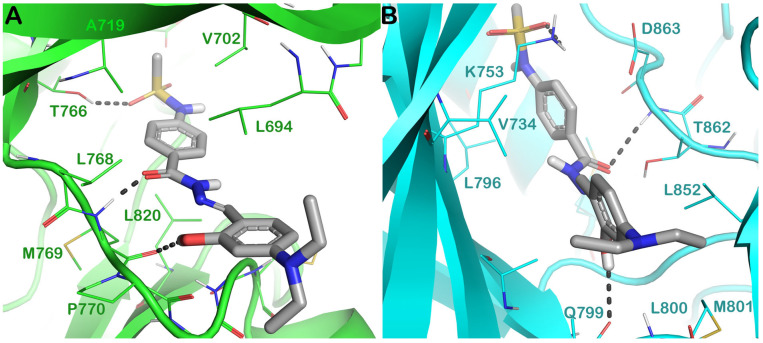
Docked poses of compound **8** (gray sticks) within the EGFR (panel (**A**), PDB ID 1M17) and HER2 (panel (**B**), PDB ID 3RCD) binding sites. The interacting residues at the binding sites are indicated by lines and are labeled. The black dotted lines represent H-bonds. The pictures were generated by PyMOL (the PyMOL Molecular Graphics System, v1.8; Schrödinger, LLC, New York, 2015).

Regarding compound **8**, the molecular docking output exhibited similar binding modes previously discussed for congeneric compound **7**, and in particular, **8,** within the EGFR binding site, could establish several polar and hydrophobic contacts (Figure 6A). The methylsulfonamide moiety formed an H-bond with the sidechain of T766. In contrast, the central region strongly targeted the backbone of residues M769 by establishing two H-bonds. Hydrophobic interactions with residues L694, V702, L820, and P770 were detected.

Accordingly, this binding mode resulted in a docking score of −7.988 kcal/mol, indicating a strong affinity of the compound to the selected binding site. Figure 6B illustrates the molecular docking output of compound **8** within the HER2 binding site. Notably, the binding mode is comparable to that of **7**. The compound strongly interacted with the binding site by targeting K753, T862, and Q799 via polar contacts. In addition, hydrophobic contacts with residues V734, L796, L800, M801, and L852 were also observed. The docking scores slightly differed from those of **7**; thus, compound **8** showed a docking score of −7.769 kcal/mol when HER2 was considered. The docking scores strongly indicated that the compounds could interact with the selected enzymes, thereby significantly inhibiting them, as observed in the in vitro tests.

**Figure 7:**
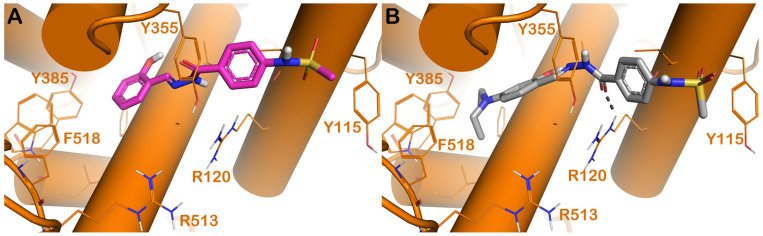
Docked poses of compound **7** (magenta sticks, panel (**A**)) and **8** (gray sticks, panel (**B**)) within the COX-2 (PDB ID 5KIR) binding sites. The interacting residues at the binding sites are indicated by lines and are labeled. The black dotted lines represent H-bonds. The pictures were generated by PyMOL (the PyMOL Molecular Graphics System, v1.8; Schrödinger, LLC, New York, 2015).

Lastly, we performed molecular docking calculations on the COX-2 enzyme to investigate the binding mode of the selected compounds to understand the limited inhibitory activity against this enzyme. Figure 7A shows the docking output of compound **7** within the COX-2 binding site. As expected, the number of contacts was particularly limited in line with the micromolar inhibition of the enzyme, as observed in the experimental assessment. In particular, we observed only hydrophobic interactions with the residues at the binding site. Compound **7** can target Y355 establishing a π-π stacking, additional hydrophobic interactions were observed with residues Y115, Y385, and F518. No other appreciable contacts were observed. This binging mode resulted in a docking score of −4.752 kcal/mol, indicating a limited affinity for the selected enzyme. Notably, the crystallized ligand rofecoxib exhibited a docking score of −9.279 kcal/mol, which is in line with the excellent inhibitory potency observed for the reference compounds. Regarding compound **8** (Figure 7B), we observed a similar binding mode with respect to compound **7** with an additional H-bond with R120 (docking score = −5.163 kcal/mol. According to the docking results, compound **8** also showed a limited inhibitory capacity against COX-2, slightly better than compound **7**. The computational assessment revealed that both compounds did not present favorable binding modes or conformational energies to guarantee strong enzyme inhibition. In fact, contrary to rofecoxib, these compounds did not show any possibility of properly targeting residues Y385, F518, and H90, which are crucial for strong binding to the COX-2 binding site.

#### 2.7.2. Molecular Dynamic Results

**Figure 8:**
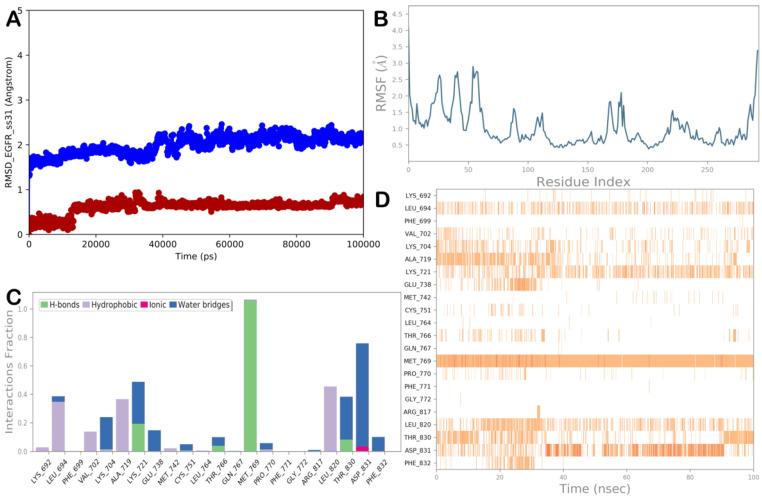
(**A**) RMSD analysis (ligand: red line; protein: blue line; compound **7** is identified by the in-house code **ss31**). (**B**) RMSF evaluation of the complex **7**/EGFR after a 100 ns MD simulation. Compound **7** is shown in (**C**,**D**) during the MD run. It is possible to distinguish between four types of interactions: H-bonds (green), ionic (magenta), hydrophobic (gray), and water bridges (blue). The stacked bar charts are standardized over the trajectory. For example, if the contact is maintained 70% of the time during the simulation, its value is 0.7. Values > 1.0 could occur if a protein residue uses the same subtype to bind with the ligand more than once. The diagram in the figure provides a temporal explanation of the main interactions. The results show residues that interact with the ligand in each trajectory frame. A deeper orange indicates several interactions between certain residues and the ligand. The images were created using software tools from Maestro and Desmond (Maestro, Schrödinger LLC, release 2020-3).

Furthermore, we validated the docking output by conducting further in silico experiments, and, in particular, we subjected the complexes obtained by the molecular docking calculations to MD simulation using the Desmond software to assess the stability of the biological systems and the stability of the retrieved binding modes. In the MD simulation experiments, we considered targets (EGFR and HER2) susceptible to the strong inhibitory effects of compounds **7** and **8**. In general, both compounds strongly bind the selected targets with high stability. Furthermore, we observed small fluctuations in the selected biological systems by analyzing the RMSD and RMSF outputs. The MD output of **7** within the EGFR binding site is presented in Figure 8. The compound maintained the binding mode identified by molecular docking for the entire duration of the MD simulation with excellent stability, as indicated by the RMSD and RMSF (Figure 8A,B).

Furthermore, the H-bonds with T766 and P770 became sporadic, whereas the H-bonds with M769 were strong and continuatively formed by **7** during the MD simulation. Interestingly, the establishment of two additional water-mediated H-bonds with T830 and D831 and an H-bond with K721 were detected. Moreover, residues L694, V702, A719, and L820 were involved in a strong network of hydrophobic interactions (Figure 8C,D). The output of the MD simulation validated the potential binding mode identified by molecular docking, indicating that **7** can inhibit EGFR.

**Figure 9:**
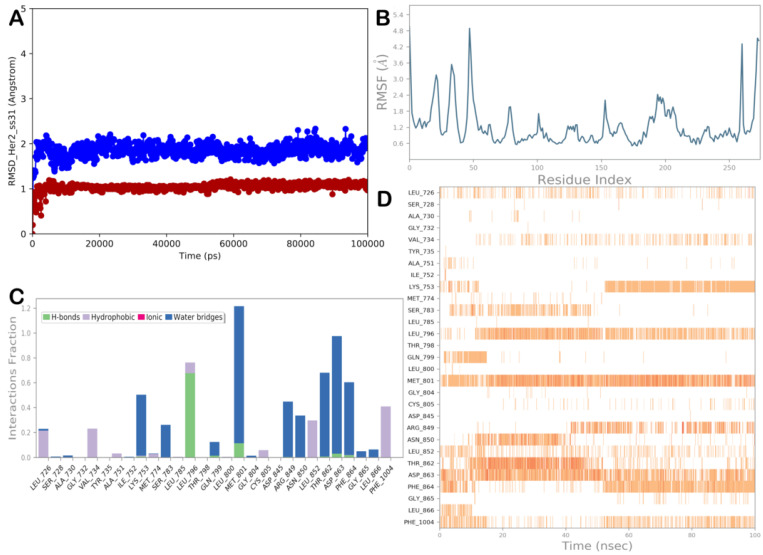
(**A**) RMSD analysis (ligand: red line; protein: blue line; compound **7** is identified by the in-house code **ss31**). (**B**) RMSF evaluation of the complex **7**/HER2 after a 100 ns MD simulation. Compound **7** is shown in (**C**,**D**) during the MD run. It is possible to distinguish between four types of interactions: H-bonds (green), ionic (magenta), hydrophobic (gray), and water bridges (blue). The stacked bar charts are standardized over the trajectory. For example, if the contact is maintained 70% of the time during the simulation, its value is 0.7. Values > 1.0 could occur if a protein residue uses the same subtype to bind with the ligand more than once. The diagram in the figure provides a temporal explanation of the main interactions. The results show residues that interact with the ligand in each trajectory frame. A deeper orange indicates several interactions between certain residues and the ligand. The images were created using software tools from Maestro and Desmond (Maestro, Schrödinger LLC, release 2020-3).

Figure 9 illustrates the MD simulation output **7** within the HER2 binding site. In this case, we also observed high stability of the biological complex, as indicated by the graphs related to RMSD and RMSF (Figure 9A,B). Furthermore, the main interactions observed in the molecular docking studies were maintained. H-bonds with K753 and T862 were maintained but became principally water-mediated, whereas H-bonds with L800 became sporadic. More favorable polar contacts were detected with the backbone of L796, whereas water-mediated H-bonds with M801, R849, N850, T862, D863, and F864 were observed. Hydrophobic interactions with residues V734, L796, and L852 were maintained. In addition, we detected further hydrophobic interactions with L726 and F1004 (Figure 9C,D), which contributed to stabilizing the proposed binding mode and exerting strong inhibitory capacity against the HER2 enzyme.

**Figure 10:**
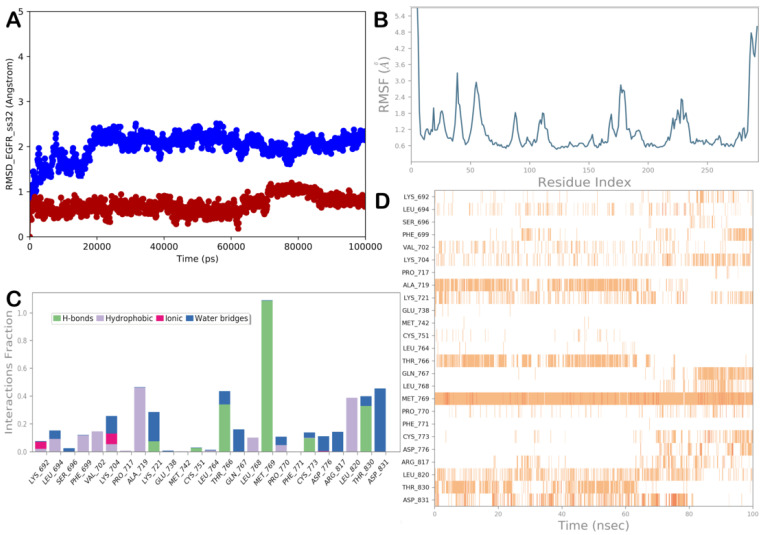
(**A**) RMSD analysis (ligand: red line; protein: blue line; compound **8** is identified by the in-house code **ss32**). (**B**) RMSF evaluation of the complex **8**/EGFR after a 100 ns MD simulation. Compound **8** is shown in (**C**,**D**) during the MD run. It is possible to distinguish between four types of interactions: H-bonds (green), ionic (magenta), hydrophobic (gray), and water bridges (blue). The stacked bar charts are standardized over the trajectory. For example, if the contact is maintained 70% of the time during the simulation, its value is 0.7. Values > 1.0 could occur if a protein residue uses the same subtype to bind with the ligand more than once. The diagram in the figure provides a temporal explanation of the main interactions. The results show residues that interact with the ligand in each trajectory frame. A deeper orange indicates several interactions between certain residues and the ligand. The images were created using software tools from Maestro and Desmond (Maestro, Schrödinger LLC, release 2020-3).

For compound **8**, the MD output of the **8**/EGFR complex is presented in Figure 10. Low RMSD and RMSF values indicate significant system stability (Figure 10A,B). Notably, the strong H-bond network with residues T766 and M769 was maintained during the 100 ns of the MD simulation. In addition, **8** formed two relevant H-bonds with T830 and D831 (water-mediated). Furthermore, additional hydrophobic interactions, over those found by molecular docking, with F699 and A719 can contribute to the tight binding of the protein, inhibiting its function (Figure 10C,D).

**Figure 11:**
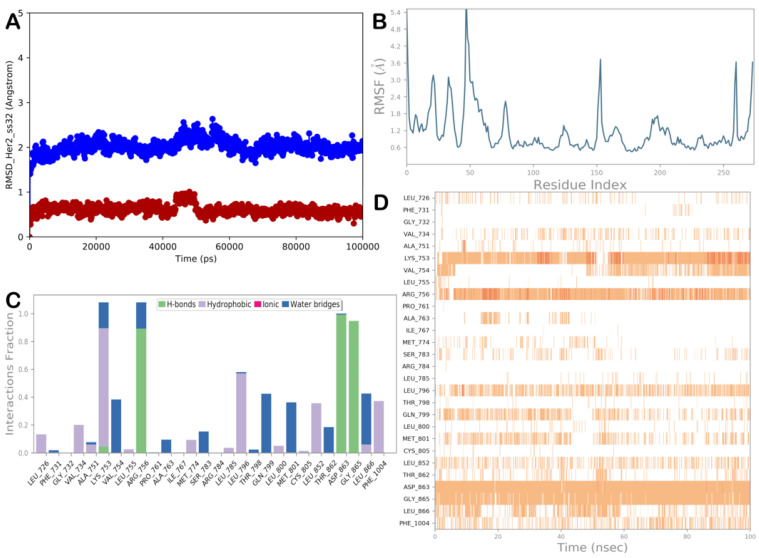
(**A**) RMSD analysis (ligand: red line; protein: blue line; compound **8** is identified by the in-house code **ss32**). (**B**) RMSF evaluation of the complex **8**/HER2 after a 100 ns MD simulation. Compound **8** is shown in (**C**,**D**) during the MD run. It is possible to distinguish between four types of interactions: H-bonds (green), ionic (magenta), hydrophobic (gray), and water bridges (blue). The stacked bar charts are standardized over the trajectory. For example, if the contact is maintained 70% of the time during the simulation, its value is 0.7. Values > 1.0 could occur if a protein residue uses the same subtype to bind with the ligand more than once. The diagram in the figure provides a temporal explanation of the main interactions. The results show residues that interact with the ligand in each trajectory frame. A deeper orange indicates several interactions between certain residues and the ligand. The images were created using software tools from Maestro and Desmond (Maestro, Schrödinger LLC, release 2020-3).

The MD output of the **8**/HER2 complex is shown in Figure 11. As observed for the other complexes, this complex was stable for 100 ns of the MD simulation (Figure 11A,B). Compound **8** maintained H-bonds with K753, T862, and Q799, although they became water-mediated, whereas more favorable polar contacts with K756, D863, and G865 were established. In addition, hydrophobic contacts with residues V734, L796, L800, M801, and L852 were maintained, and additional contacts were observed with K753 and F1004 (Figure 11C,D). Accordingly, the computational output confirmed the strong potential of **8** against HER2.

## 3. Conclusions

Five Schiff bases (**7**–**11**) containing methanesulfonamide were synthesized and tested for their antitumor properties against 59 different cell lines at a concentration of 10 µM. Derivatives **7** and **8** showed the most potent antitumor action, with positive cytotoxic effects of 54/59 and 59/59 and mean growth percentages of 67.3% and 19.5%, respectively, compared with imatinib (PCE = 20/59 and MG% = 92.6%). Schiff base **8** was the most potent antitumor agent among the five evaluated compounds, with the highest potency in suppressing cell proliferation by an average of 50% at a dosage of 0.501 µM. The assay results were compared with sorafenib, gefitinib, erlotinib, and celecoxib potencies. The antitumor effect of derivative **8** was five-fold higher than that of sorafenib (MG_MID = 2.33 µM), four-fold higher than gefitinib (MG_MID = 2.1 µM), fifteen-fold higher than erlotinib (MG_MID = 7.68 µM), and thirty-four-fold higher than celecoxib (MG_MID = 17.5 µM). Schiff bases **7** and **8** inhibited the EGFR/HER2 kinase and COX-2 enzymes. They exhibited significant HER2 inhibition at IC_50_ values of 0.183 μM and 0.464 μM. Their EGFR inhibition was also substantial, as evidenced by their respective IC_50_ values of 0.166 μM and 0.752 μM. Compound **8**, with an IC_50_ of 12.76 μM, had the highest COX-2 inhibition. The effects of compounds **7** and **8** on MCF-7 cells on Bax, Bcl-2, caspase-8, and caspase-9 were examined. The results showed that compounds **7** and **8** raised the levels of caspase-8 and caspase-9 in MCF-7 cells compared to standard drugs lapatinib and staurosporine. They also raised the levels of Bax protein expression and lowered the levels of Bcl-2 protein expression. Also, compound **8** interfered with the normal cell cycle, increasing the percentage of apoptotic cells and causing cell cycle arrest. Compounds **7** and **8** were computationally investigated using molecular docking and molecular dynamics in the binding sites of EGFR and HER2 to determine the binding mode and molecular features necessary for these compounds’ interaction with the corresponding enzymes or receptors.

## 4. Experimental

### 4.1. Chemistry

Following our previous study, we synthesized Schiff bases **7**–**11** [66]. We identified the chemical structures using NMR, IR, and mass spectroscopy.

### 4.2. Biological Evaluation

#### 4.2.1. In Vitro Antitumor Assay

The Drug Evaluation Branch of the National Cancer Institute, Bethesda, MD, USA, performed the antitumor assay using fifty-nine human tumor cell lines from nine human tissues. As described in our previous reports, we calculate compound **8**’s three dose–response parameters—GI_50_, TGI, and LC_50_ (Supplementary Materials) [68]. 

#### 4.2.2. In Vitro Cyclooxygenase (COX) Inhibition Assay

Following the guidelines provided by the manufacturer’s instructions, we employed the colorimetric COX-2 inhibition assay (kit catalog number 560101, Cayman Chemical, Ann Arbour, MI, USA) to assess the inhibitory power of celecoxib and the evaluated derivatives on the COX-2 isozyme (Supplementary Materials) [69].

#### 4.2.3. EGFR and HER2 Tyrosine Kinases Assay

As mentioned in the previous reports, we performed the EGFR and HER2 enzyme inhibition assays. In the experiments, we used the most potent antitumor, Schiff bases **7** and **8**, to inhibit the EGFR and HER2 enzymes (Supplementary Materials) [47,61].

#### 4.2.4. Apoptosis Assay

Following our previous study, we used the MCF-7 cell line and a well-known Annexin 5-FITC/PI detection kit to induce apoptosis. The FACSCalibur flow cytometer was used to analyze the cell line samples (Supplementary Materials) [47,71].

#### 4.2.5. Cell Cycle Analysis

Using the MCF-7 cell line, the cell cycle analysis was carried out like our previous investigation. A FACSCalibur flow cytometer was used to analyze the cells after they had been stained with DNA fluorochrome PI (Supplementary Materials) [47,72].

#### 4.2.6. Apoptotic Markers Assay

The apoptotic marker method was carried out using the reported procedure using MCF-7, the cancer cell line (Supplementary Materials) [32]. 

### 4.3. Molecular Docking Computation

Compounds **7** and **8** were developed in Maestro (Maestro, Schrödinger, LLC, New York, NY, USA, 2020, release 3, version 12.5) using dedicated drawing tools. The compounds were minimized using OPLS3 as a force field [74] (MacroModel, Schrödinger, LLC, New York, NY, USA, 2020, version 12.9) with GB/SA as the solvation model to simulate the solvent effect (no cutoff was utilized). The PRCG method (5000 maximum iterations) was utilized with a gradient convergence threshold of 0.001. The resulting structures were treated using LigPrep (LigPrep, Schrödinger, LLC, New York, NY, USA, 2020, version 2.5) to obtain the potential ionization states of **7** and **8** at physiological pH (7.4 ± 0.2) and to prevent possible errors in the corresponding chemical structures [75]. The three-dimensional structures of the target enzymes *h*EGFR, *h*HER2, and *h*COX-2 were downloaded from the protein data bank (PDB IDs 1M17, 3RCD, and 5KIR, respectively). The biological complexes were imported into Maestro, and after removing the substances employed in the crystallization procedure, they were treated utilizing the Protein Preparation Wizard implemented in Maestro Suite, as previously described [76,77], to obtain suitable initial structures for further in silico investigations. Molecular docking calculations were performed by the Glide software (Glide, Schrödinger, LLC, New York, NY, USA, 2020, version 8.8) and standard precision (SP) was used [78,79]. Energy grids were built using a cubic box centered on the crystallized inhibitors (atom scaling factor 1.0 Å) with default settings without constraint. Compounds were docked, and 50 poses were included in the post-docking minimization step.

### 4.4. Molecular Dynamic Methodology

The MD simulation studies were conducted using the Desmond software with Maestro 12.6 (Desmond Molecular Dynamics System 6.4 academic version, D. E. Shaw Research (“DESRES”), New York, NY, USA, 2020. Maestro-Desmond Interoperability Tools, Schrödinger, New York, NY, USA, 2020). Suitable biological systems (**7**/*h*EGFR, **7**/*h*HER2, **8**/*h*EGFR, and **8/***h*HER2) were obtained using the system builder tool provided by Desmond. Via this tool, the ligand/protein structures were positioned in an orthorhombic box and solvated with water molecules (TIP3P) at a salt physiological concentration of 0.15 M (Na^+^ and Cl^−^ ions were added) [80,81]. The CUDA API technology on two NVIDIA graphics processing units (GPUs) was employed to perform the MD calculations utilizing the OPLS force field [74]. The MD simulation studies were conducted by using the ensemble class NPT (constant number of particles, pressure (1.01325 bar) and temperature (300 K)) with the RESPA integrator [82]. The temperature and pressure were maintained constant by the Nose–Hoover thermostat technique [83] and the Martyna–Tobias–Klein method [84]. The particle-mesh Ewald technique (PME) was used to compute long-range electrostatic interactions, whereas van der Waals and short-range electrostatic interactions were settled at 9.0 Å [85]. Each system was gradually relaxed and brought to equilibrium using a default procedure that included several constrained minimizations and MD simulations. The simulation analysis tools in Desmond were used to analyze the MD outputs.

## Figures and Tables

**Table 1:** Antitumor activity of pyrazolines **7**–**11** presented as growth inhibition percentages (GI%) using 59 subpanel tumor cell lines.

Compd No.	PCE *	Cancer Cell Line Assays (10.0 µM in One Dose, GI%) **	MG% ***
**7**	54/59	*Leukemia* (CCRF-CEM, 63%; HL-60(TB), 22%; K-562, 26%; MOLT-4, 50%; SR, 67%), *NSC Lung* (A549/ATCC, 11%; EKVX, 26%; HOP-62, 61%; HOP-92, 44%; NCI-H226, 25%; NCI-H23, 27%; NCI-H322M, 54%; NCI-H460, 21%), *Colon* (HCC-2998, 41%; HCT-116, 59%; HCT-15, 67%; KM12, 35%; SW-620, 35%), *CNS* (SF-268, 48%; SF-295, 35%; SF-539, 40%; SNB-19, 32%; U251, 56%), *Melanoma* (LOX IMVI, 53%; MALME-3M, 24%; M14, 44%; MDA-MB-435, 24%; SK-MEL-2, 22%; SK-MEL-28, 37%; SK-MEL-5, 33%; UACC-62, 35%), *Ovarian* (IGROV1, 25%; OVCAR-4, 53%; OVCAR-5, 23%; OVCAR-8, 32%; NCI/ADR-RES, 41%; SK-OV-3, 42%), *Renal* (786-0, 25%; A498, 16%; ACHN, 46%; CAKI-1, 35%; RXF 393, 21%; SN12C, 23%; TK-10, 21%; UO-31, 64%), *Prostate* (PC-3, 38%; DU-145, 33%), *Breast* (MCF-7, 21%; MDA-MB-231/ATCC, 34%; HS 578T, 11%; BT-549, 27%; T-47D, 18%; MDA-MB-468, 24%).	67.32
**8**	59/59	*Leukemia* (CCRF-CEM, 94%; HL-60(TB), 99%; K-562, 86%; MOLT-4, 90%; RPMI-8226, 84%; SR, 89%), *NSC Lung* (A549/ATCC, 88%; EKVX, 84%; HOP-62, 87%; HOP-92, 61%; NCI-H226, 61%; NCI-H23, 77%; NCI-H322M, 75%; NCI-H460, 97%; NCI-H522, 88%), *Colon* (COLO 205, 94%; HCC-2998, 62%; HCT-116, 78%; HCT-15, 80%; HT29, 73%; KM12, 85%; SW-620, 63%), *CNS* (SF-268, 77%; SF-295, 90%; SF-539, 93%; SNB-19, 65%; SNB-75, 52%; U251, 76%), *Melanoma* (LOX IMVI, 100%; MALME-3M,96%; M14, 100%; MDA-MB-435, 71%; SK-MEL-2, 59%; SK-MEL-28, 77%; SK-MEL-5, 100%; UACC-257, >100%; UACC-62, 100%), *Ovarian* (IGROV1, 80%; OVCAR-4, 69%; OVCAR-5, 54%; OVCAR-8, 88%; NCI/ADR-RES, 63%; SK-OV-3, 66%), *Renal* (786-0, 98%; A498, 81%; ACHN, 84%; CAKI-1, 81%; RXF 393, >100%; SN12C, 69%; TK-10, 52%; UO-31, 87%), *Prostate* (PC-3, 68%; DU-145, 69%), *Breast* (MCF-7, 94%; MDA-MB-231/ATCC, 62%; HS 578T, 44%; BT-549, 88%; T-47D, 74%; MDA-MB-468, >100%).	19.55
**9**	3/59	*NSC Lung* (HOP-92, 23%), *Melanoma* (MALME-3M, 12%), *Renal* (UO-31, 17%).	97.67
**10**	4/59	*Melanoma* (UACC-257, 10%), *Renal* (A498, 15%; CAKI-1, 10%; UO-31, 14%).	100.00
**11**	4/59	*Leukemia* (SR, 10%), *Renal* (CAKI-1, 16%; UO-31, 10%), *Breast* (MCF-7, 11%).	101.84
**Imatinib**	20/59	*Leukemia* (MOLT-4, 18%; PRMI-8226, 13%; SR, 15%), *NSC Lung* (EKVX, 16%; NCI-H226, 11%; NCI-H23, 17%), *Colon* (HCT-116, 19%; HCT-15, 12%; HT-29, 47%), *CNS* (SF-295, 15%; SF-539, 25%; U251, 11%), *Melanoma* (LOX IMVI, 12%; SK-MEL-5, 22%), *Renal* (A-498, 14%), *Prostate* (PC-3, 11%; DU-145, 14%), *Breast* (MDA-MB-231/ATCC, 11%; T-47D, 19%; MDA-MB-468, 29%).	92.62

* PCE: Positive cytotoxic effect; the ratio between the number of cell lines with percentage growth inhibition > 10% and the total cell lines. ** GI%: Growth Inhibition Percent. *** MG%: Mean growth percent.

**Table 2:** Influence of compound **8** and reference drugs on the growth of the individual tumor cell lines; median growth inhibitory (GI_50_, μM).

Subpanel Tumor Cell Lines	GI_50_ (μM)				
	8 (D-830259/1) ^1^	Sorafenib (747971) ^1^	Gefitinib (759856) ^1^	Erlotinib (718781) ^1^	Celecoxib (719627) ^1^
*Leukemia*					
CCRF-CEM	0.177	1.99	0.398	50.12	12.58
HL-60(TB)	0.30	1.58	1.0	25.12	15.85
K-562	0.492	3.16	2.51	31.62	10.00
MOLT-4	0.372	3.16	1.995	25.12	15.85
PRMI-8226	2.72	1.58	6.31	25.12	3.98
SR	0.141	3.16	3.16	10.00	15.85
*Non-Small Cell Lung Cancer*					
A549/ATCC	1.34	3.16	2.51	10.00	15.85
EKVX	0.386	2.51	0.0501	0.199	19.95
HOP-62	0.189	1.99	3.16	15.85	63.09
HOP-92	0.443	1.58	0.316	3.98	NT ^2^
NCI-H226	0.986	1.99	3.98	39.81	NT ^2^
NCI-H23	1.16	1.99	2.51	31.62	15.85
NCI-H322M	0.258	2.51	0.063	0.10	19.95
NCI-H460	0.475	2.51	3.16	6.31	15.85
NCI-H522	0.36	1.99	1.0	1.00	NT ^2^
*Colon Cancer*					
COLO 205	0.349	1.99	7.94	50.12	15.85
HCC-2998	0.356	3.16	2.51	79.43	15.85
HCT-116	0.263	1.58	3.16	6.31	50.01
HCT-15	0.358	2.51	3.98	3.98	15.85
HT29	0.495	1.99	3.98	63.09	15.85
KM12	0.509	1.58	10.0	79.43	15.85
SW-620	0.471	2.51	5.011	79.43	15.85
*CNS Cancer*					
SF-268	0.252	2.51	2.51	15.84	15.85
SF-295	0.517	1.58	10.0	15.84	15.85
SF-539	0.362	1.58	10.0	19.95	19.95
SNB-19	0.831	3.16	3.16	12.58	19.95
SNB-75	0.519	3.16	5.011	12.58	15.85
U251	0.226	1.99	3.16	25.12	15.85
*Melanoma*					
LOX IMVI	0.209	1.58	3.16	6.31	19.95
MALME-3M	0.855	1.99	3.98	1.58	19.95
M14	0.351	1.99	1.995	6.31	63.09
MDA-MB-435	0.558	1.58	3.16	19.95	15.85
SK-MEL-2	0.749	1.99	10.0	6.31	15.85
SK-MEL-28	0.381	2.51	3.16	50.12	15.85
SK-MEL-5	1.13	1.58	3.16	19.5	15.85
UACC-257	0.463	1.99	2.51	100	19.95
UACC-62	0.375	1.58	1.995	3.16	15.85
*Ovarian Cancer*					
IGROV1	0.544	2.51	3.16	0.316	15.85
OVCAR-4	0.388	3.16	6.31	10.00	12.58
OVCAR-5	1.89	3.16	3.98	7.94	19.95
OVCAR-8	0.312	3.16	0.631	7.94	19.95
NCI/ADR-RES	2.58	2.51	1.26	7.94	15.85
SK-OV-3	0.45	2.51	1.0	0.50	19.95
*Renal Cancer*					
786-0	0.358	3.16	3.16	10.00	39.81
A498	2.23	2.51	0.631	2.51	15.85
ACHN	0.513	2.51	0.158	0.199	15.85
CAKI-1	0.464	3.16	0.5012	0.199	19.95
RXF 393	1.04	2.51	3.16	3.98	15.85
SN12C	0.603	2.51	1.258	1.58	15.85
TK-10	1.05	3.98	0.794	0.251	19.95
UO-31	0.346	2.51	1.584	1.00	15.85
*Prostate Cancer*					
PC-3	0.368	1.99	5.011	39.81	12.58
DU-145	0.685	3.16	1.584	1.99	15.85
*Breast Cancer*					
MCF-7	0.416	2.51	5.011	100.00	15.85
MDA-MB-231/ATCC	0.425	1.26	3.98	6.31	15.85
HS 578T	1.47	2.51	7.94	6.31	19.95
BT-549	0.569	3.16	3.16	31.62	19.95
T-47D	0.382	1.58	7.94	3.98	15.85
MDA-MB-468	0.335	1.99	0.01258	0.126	NT ^2^

^1^ https://dtp.cancer.gov/dtpstandard/dwindex/index.jsp (accessed on 6 May 2024 ). ^2^ NT: Not tested.

**Table 3:** Average antitumor activity of compound **8** and reference drugs against tumor cell lines from nine different organs at 10-fold dilution of five concentrations; median growth inhibitory (GI_50_, μM), total growth inhibitory (TGI, μM), and median lethal (LC_50_, μM) ^1^.

	Subpanel Tumor Cell Lines										MG-MID ^2^
Compd. No. (NSC) ^3^	Activity	Leukemia	NSC Lung Cancer	Colon Cancer	CNS Cancer	Melanoma	Ovarian Cancer	Renal Cancer	Prostate Cancer	Breast Cancer	
**8** (799156)	GI_50_	0.70	0.622	0.40	0.451	0.563	1.03	0.826	0.527	0.599	0.501
	TGI	79.88	22.08	30.53	14.14	21.64	52.90	23.37	60.80	15.22	46.8
	LC_50_	c	94.94	80.1	86.63	93.63	94.88	80.60	97.95	99.85	c
Sorafenib (747971)	GI_50_	1.91	2.34	2.19	2.33	1.87	2.89	2.86	2.58	2.17	2.33
	TGI	42.12	7.99	7.94	7.64	6.29	14.43	10.86	10.27	9.35	9.11
	LC_50_	c	49.15	38.06	27.77	28.57	71.34	54.75	69.91	57.29	43.1
Gefitinib (759856)	GI_50_	2.56	2.05	5.23	5.64	3.68	3.05	1.41	3.29	4.67	2.1
	TGI	12.07	13.86	18.47	19.62	12.49	33.29	12.50	31.62	18.62	14.3
	LC_50_	93.85	94.68	51.74	50.56	36.40	83.58	52.82	89.72	52.47	51.9
Erlotinib (718781)	GI_50_	27.85	13.11	51.68	16.99	23.74	5.52	2.46	20.90	24.72	7.68
	TGI	96.57	73.76	c	82.11	77.89	74.41	42.59	c	70.53	66.3
	LC_50_	c	97.71	c	c	93.31	97.06	89.15	c	96.43	95.6
Celecoxib (719627)	GI_50_	12.35	25.09	20.73	17.22	22.46	17.14	19.87	14.22	17.49	17.5
	TGI	29.27	41.93	41.39	31.62	38.49	34.20	41.19	28.37	34.89	34.0
	LC_50_	67.09	67.08	66.51	58.76	64.31	65.91	61.22	57.01	67/03	63.3

^1^ GI_50_, the molar concentration of the compound that inhibits 50% net cell growth; TGI, the molar concentration of the compound leading to total inhibition; and LC_50_, the molar concentration of the compounds leading to 50% net cell death. ^2^ Full panel mean-graph midpoint (μM). ^3^ https://dtp.cancer.gov/dtpstandard/dwindex/index.jsp (accessed on 6 May 2024). c: compounds showed values ≥ 100 μM.

**Table 4:** In vitro inhibitory effects of EGFR, HER2, and COX−2 of the antitumor agents.

Compound No.	IC_50_ (μM)		
	EGFR Inhibition	HER2 Inhibition	COX−2 Inhibition
**7**	0.752 ± 0.05	0.183 ± 0.011	20.86 ± 0.82
**8**	0.166 ± 0.033	0.464 ± 0.018	12.76 ± 0.51
Erlotinib	0.105 ± 0.035	0.085 ± 0.003	-
Lapatinib	0.045 ± 0.002	0.033 ± 0.001	-
Ibuprofen	-	-	3.065 ± 0.12
Celecoxib	-	-	2.900 ± 0.10

**Table 5:** Effect of compound **8** on the percentage of MCF-7 cells stained positive for annexin V-FITC and on the cell cycle of MCF-7 cells.

Sample	Total Apoptosis	Early Apoptosis	Late Apoptosis	Necrosis
**8**	38.16	22.49	9.59	6.08
Staurosporine	34.39	8.36	21.99	4.04
DMSO	2.09	0.44	0.16	1.49
	**%G0–G1**	**%S**	**%G2-M**	**Comment**
**8**	51.94	33.42	14.64	Growth arrest@ S
Staurosporine	65.74	31.19	3.07	Growth arrest@G1/S
DMSO	55.81	26.76	17.43	

**Table 6:** Effect of compounds **7** and **8** and DMSO on *Bax*, *Bcl-2*, and *Caspase-3*/*-9* levels inside MCF-7 cells treated with the compounds at IC_50_ concentrations.

Gene Expression (Fold Change)				
Compound No.	*Bax*	*Bcl2*	*Casp8*	*Casp9*
**7**	5.02	0.201	5.171	2.990
**8**	5.456	0.232	6.456	3.776
Lapatinib	7.224	0.278	2.887	6.745
Staurosporine	5.987	0.287	7.342	5.034
Control	1	1	1	1

## Data Availability

All data are included within the manuscript and Supplementary Materials.

## Supplementary Materials

The following supporting information can be downloaded at https://www.mdpi.com/article/10.3390/ijms252413621/s1. References [47,68,71,72,86,87,88,89,90,91,92,93,94,95,96,97] are cited in Supplementary Materials file.

## References

[1] Bray F., Laversanne M., Sung H., Ferlay J., Siegel R.L., Soerjomataram I., Jemal A. (2024). Global cancer statistics 2022: GLOBOCAN estimates of incidence and mortality worldwide for 36 cancers in 185 countries. C A Cancer J. Clin..

[2] Vainshelboim B., Müller J., Lima R.M., Nead K.T., Chester C., Chan K., Kokkinos P., Myers J. (2017). Cardiorespiratory fitness, physical activity and cancer mortality in men. Prev. Med..

[3] Szakács G., Paterson J.K., Ludwig J.A., Booth-Genthe C., Gottesman M.M. (2006). Targeting multidrug resistance in cancer. Nat. Rev. Drug Discov..

[4] El-Ayaan U., Abdel-Aziz A.A.-M., Al-Shihry S. (2007). Solvatochromism, DNA binding, antitumor activity and molecular modeling study of mixed-ligand copper(II) complexes containing the bulky ligand: Bis[N-(p-tolyl)imino]acenaphthene. Eur. J. Med. Chem..

[5] Turky A., Bayoumi A.H., Ghiaty A., El-Azab A.S., Abdel-Aziz A.A.-M., Abulkhair H.S. (2020). Design, synthesis, and antitumor activity of novel compounds based on 1,2,4-triazolophthalazine scaffold: Apoptosis-inductive and PCAF-inhibitory effects. Bioorg. Chem..

[6] El-Husseiny W.M., El-Sayed M.A., Abdel-Aziz N.I., El-Azab A.S., Asiri Y.A., Abdel-Aziz A.A.-M. (2018). Structural alterations based on naproxen scaffold: Synthesis, evaluation of antitumor activity and COX-2 inhibition, and molecular docking. Eur. J. Med. Chem..

[7] El-Sherbeny M.A., Abdel-Aziz A.A.-M., Ahmed M.A. (2010). Synthesis and antitumor evaluation of novel diarylsulfonylurea derivatives: Molecular modeling applications. Eur. J. Med. Chem..

[8] El-Deeb I.M., Bayoumi S.M., El-Sherbeny M.A., Abdel-Aziz A.A.-M. (2010). Synthesis and antitumor evaluation of novel cyclic arylsulfonylureas: ADME-T and pharmacophore prediction. Eur. J. Med. Chem..

[9] El-Azab A.S., Alanazi A.M., Abdel-Aziz N.I., Al-Suwaidan I.A., El-Sayed M.A., El-Sherbeny M.A., Abdel-Aziz A.A.-M. (2013). Synthesis, molecular modeling study, preliminary antibacterial, and antitumor evaluation of N-substituted naphthalimides and their structural analogues. Med. Chem. Res..

[10] Alanazi A.M., Abdel-Aziz A.A.-M., Shawer T.Z., Ayyad R.R., Al-Obaid A.M., Al-Agamy M.H., Maarouf A.R., El-Azab A.S. (2016). Synthesis, antitumor and antimicrobial activity of some new 6-methyl-3-phenyl-4(3H)-quinazolinone analogues: In silico studies. J. Enzyme Inhib. Med. Chem..

[11] Alanazi A.M., Abdel-Aziz A.A.-M., Al-Suwaidan I.A., Abdel-Hamide S.G., Shawer T.Z., El-Azab A.S. (2014). Design, synthesis and biological evaluation of some novel substituted quinazolines as antitumor agents. Eur. J. Med. Chem..

[12] El-Azab A.S., Abdel-Aziz A.A.-M., Ghabbour H.A., Al-Gendy M.A. (2017). Synthesis, *in vitro* antitumour activity, and molecular docking study of novel 2-substituted mercapto-3-(3,4,5-trimethoxybenzyl)-4(3H)-quinazolinone analogues. J. Enzyme Inhib. Med. Chem..

[13] El-Azab A.S., Al-Dhfyan A., Abdel-Aziz A.A.-M., Abou-Zeid L.A., Alkahtani H.M., Al-Obaid A.M., Al-Gendy M.A. (2017). Synthesis, anticancer and apoptosis-inducing activities of quinazoline-isatin conjugates: Epidermal growth factor receptor-tyrosine kinase assay and molecular docking studies. J. Enzyme Inhib. Med. Chem..

[14] Mohamed M.A., Ayyad R.R., Shawer T.Z., Abdel-Aziz A.A.-M., El-Azab A.S. (2016). Synthesis and antitumor evaluation of trimethoxyanilides based on 4(3H)-quinazolinone scaffolds. Eur. J. Med. Chem..

[15] El-Sayed M.A., El-Husseiny W.M., Abdel-Aziz N.I., El-Azab A.S., Abuelizz H.A., Abdel-Aziz A.A.-M. (2018). Synthesis and biological evaluation of 2-styrylquinolines as antitumour agents and EGFR kinase inhibitors: Molecular docking study. J. Enzyme Inhib. Med. Chem..

[16] Abdel-Aziz A.A.-M., El-Azab A.S., El-Subbagh H.I., Al-Obaid A.M., Alanazi A.M., Al-Omar M.A. (2012). Design, synthesis, single-crystal and preliminary antitumor activity of novel arenesulfonylimidazolidin-2-ones. Bioorg Med. Chem. Lett..

[17] Al-Suwaidan I.A., Alanazi A.M., Abdel-Aziz A.A.-M., Mohamed M.A., El-Azab A.S. (2013). Design, synthesis and biological evaluation of 2-mercapto-3-phenethylquinazoline bearing anilide fragments as potential antitumor agents: Molecular docking study. Bioorg Med. Chem. Lett..

[18] Alanazi A.M., Al-Suwaidan I.A., Abdel-Aziz A.A.-M., Mohamed M.A., El_Morsy A.M., El-Azab A.S. (2013). Design, synthesis and biological evaluation of some novel substituted 2-mercapto-3-phenethylquinazolines as antitumor agents. Med. Chem. Res..

[19] Abdel-Aziz A.A.-M., El-Azab A.S., Alanazi A.M., Asiri Y.A., Al-Suwaidan I.A., Maarouf A.R., Ayyad R.R., Shawer T.Z. (2016). Synthesis and potential antitumor activity of 7-(4-substituted piperazin-1-yl)-4-oxoquinolines based on ciprofloxacin and norfloxacin scaffolds: In silico studies. J. Enzyme Inhib. Med. Chem..

[20] Hamdi A., El-Shafey H.W., Othman D.I.A., El-Azab A.S., AlSaif N.A., Abdel-Aziz A.A.-M. (2022). Design, synthesis, antitumor, and VEGFR-2 inhibition activities of novel 4-anilino-2-vinyl-quinazolines: Molecular modeling studies. Bioorg. Chem..

[21] El-Husseiny W.M., El-Sayed M.A., Abdel-Aziz N.I., El-Azab A.S., Ahmed E.R., Abdel-Aziz A.A.-M. (2018). Synthesis, antitumour and antioxidant activities of novel α,β-unsaturated ketones and related heterocyclic analogues: EGFR inhibition and molecular modelling study. J. Enzyme Inhib. Med. Chem..

[22] Alanazi A.M., El-Azab A.S., Al-Swaidan I.A., Maarouf A.R., El-Bendary E.R., El-Enin M.A.A., Abdel-Aziz A.A.-M. (2013). Synthesis, single-crystal, *in vitro* antitumor evaluation and molecular docking of 3-substitued 5, 5-diphenylimidazolidine-2, 4-dione derivatives. Med. Chem. Res..

[23] Al-Suwaidan I.A., Abdel-Aziz A.A.-M., Shawer T.Z., Ayyad R.R., Alanazi A.M., El-Morsy A.M., Mohamed M.A., Abdel-Aziz N.I., El-Sayed M.A., El-Azab A.S. (2016). Synthesis, antitumor activity and molecular docking study of some novel 3-benzyl-4(3H)quinazolinone analogues. J. Enzyme Inhib. Med. Chem..

[24] Bayat Mokhtari R., Homayouni T.S., Baluch N., Morgatskaya E., Kumar S., Das B., Yeger H. (2017). Combination therapy in combating cancer. Oncotarget.

[25] Stratikopoulos E.E., Dendy M., Szabolcs M., Khaykin A.J., Lefebvre C., Zhou M.M., Parsons R. (2015). Kinase and BET Inhibitors Together Clamp Inhibition of PI3K Signaling and Overcome Resistance to Therapy. Cancer Cell..

[26] Fu R.G., Sun Y., Sheng W.B., Liao D.F. (2017). Designing multi-targeted agents: An emerging anticancer drug discovery paradigm. Eur. J. Med. Chem..

[27] Zhang L., Dewan V., Yin H. (2017). Discovery of Small Molecules as Multi-Toll-like Receptor Agonists with Proinflammatory and Anticancer Activities. J. Med. Chem..

[28] El-Azab A.S., Abdel-Aziz A.A.-M., Abou-Zeid L.A., El-Husseiny W.M., El Morsy A.M., El-Gendy M.A., El-Sayed M.A. (2018). Synthesis, antitumour activities and molecular docking of thiocarboxylic acid ester-based NSAID scaffolds: COX-2 inhibition and mechanistic studies. J. Enzyme Inhib. Med. Chem..

[29] Alkahtani H.M., Abdalla A.N., Obaidullah A.J., Alanazi M.M., Almehizia A.A., Alanazi M.G., Ahmed A.Y., Alwassil O.I., Darwish H.W., Abdel-Aziz A.A.-M. (2020). Synthesis, cytotoxic evaluation, and molecular docking studies of novel quinazoline derivatives with benzenesulfonamide and anilide tails: Dual inhibitors of EGFR/HER2. Bioorg Chem..

[30] Daydé-Cazals B., Fauvel B., Singer M., Feneyrolles C., Bestgen B., Gassiot F., Spenlinhauer A., Warnault P., Van Hijfte N., Borjini N. (2016). Rational Design, Synthesis, and Biological Evaluation of 7-Azaindole Derivatives as Potent Focused Multi-Targeted Kinase Inhibitors. J. Med. Chem..

[31] Abdel-Aziz A.A.-M., Angeli A., El-Azab A.S., Hammouda M.E.A., El-Sherbeny M.A., Supuran C.T. (2019). Synthesis and anti-inflammatory activity of sulfonamides and carboxylates incorporating trimellitimides: Dual cyclooxygenase/carbonic anhydrase inhibitory actions. Bioorg Chem..

[32] Abdel-Aziz A.A.-M., El-Azab A.S., Brogi S., Ayyad R.R., Alkahtani H.M., Abuelizz H.A., Al-Suwaidan I.A., Al-Obaid A.M. (2024). Synthesis, enzyme inhibition assay, and molecular modeling study of novel pyrazolines linked to 4-methylsulfonylphenyl scaffold: Antitumor activity and cell cycle analysis. RSC Adv..

[33] Alkahtani H.M., Alanazi M.M., Aleanizy F.S., Alqahtani F.Y., Alhoshani A., Alanazi F.E., Almehizia A.A., Abdalla A.N., Alanazi M.G., El-Azab A.S. (2019). Synthesis, anticancer, apoptosis-inducing activities and EGFR and VEGFR2 assay mechanistic studies of 5,5-diphenylimidazolidine-2,4-dione derivatives: Molecular docking studies. Saudi Pharm. J..

[34] Hamdi A., Tawfik S.S., Ali A.R., Ewes W.A., Haikal A., El-Azab A.S., Bakheit A.H., Hefnawy M.M., Ghabbour. H.A., Abdel-Aziz A.A.-M. (2024). Harnessing potential COX-2 engagement for boosting anticancer activity of substituted 2-mercapto-4(3H)-quinazolinones with promising EGFR/VEGFR-2 inhibitory activities. Bioorg Chem..

[35] Ishikawa T., Seto M., Banno H., Kawakita Y., Oorui M., Taniguchi T., Ohta Y., Tamura T., Nakayama A., Miki H. (2011). Design and synthesis of novel human epidermal growth factor receptor 2 (HER2)/epidermal growth factor receptor (EGFR) dual inhibitors bearing a pyrrolo[3,2-d]pyrimidine scaffold. J. Med. Chem..

[36] Kolibaba K.S., Druker B.J. (1997). Protein tyrosine kinases and cancer. Biochim. Biophys. Acta.

[37] Iqbal N., Iqbal N. (2014). Human Epidermal Growth Factor Receptor 2 (HER2) in Cancers: Overexpression and Therapeutic Implications. Mol. Biol. Int..

[38] Vranić S., Bešlija S., Gatalica Z. (2021). Targeting HER2 expression in cancer: New drugs and new indications. Bosn. J. Basic. Med. Sci..

[39] Hamilton E., Shastry M., Shiller S.M., Ren R. (2021). Targeting HER2 heterogeneity in breast cancer. Cancer Treat. Rev..

[40] Daks A.A., Fedorova O.A., Shuvalov O.Y., Parfenev S.E., Barlev N.A. (2020). The Role of ERBB2/HER2 Tyrosine Kinase Receptor in the Regulation of Cell Death. Biochemistry.

[41] Wee P., Wang Z. (2017). Epidermal Growth Factor Receptor Cell Proliferation Signaling Pathways. Cancers.

[42] Woodburn J.R. (1999). The epidermal growth factor receptor and its inhibition in cancer therapy. Pharmacol. Ther..

[43] Salomon D.S., Brandt R., Ciardiello F., Normanno N. (1995). Epidermal growth factor-related peptides and their receptors in human malignancies. Crit. Rev. Oncol. Hematol..

[44] Antonello A., Tarozzi A., Morroni F., Cavalli A., Rosini M., Hrelia P., Bolognesi M.L., Melchiorre C. (2006). Multitarget-directed drug design strategy: A novel molecule designed to block epidermal growth factor receptor (EGFR) and to exert proapoptotic effects. J. Med. Chem..

[45] Madhusudan S., Ganesan T.S. (2004). Tyrosine kinase inhibitors in cancer therapy. Clin. Biochem..

[46] Xia X., Gong C., Zhang Y., Xiong H. (2023). The History and Development of HER2 Inhibitors. Pharmaceuticals.

[47] El-Azab A.S., Abdel-Aziz A.A.-M., AlSaif N.A., Alkahtani H.M., Alanazi M.M., Obaidullah A.J., Eskandrani R.O., Alharbi A. (2020). Antitumor activity, multitarget mechanisms, and molecular docking studies of quinazoline derivatives based on a benzenesulfonamide scaffold: Cell cycle analysis. Bioorg Chem..

[48] Xu G., Abad M.C., Connolly P.J., Neeper M.P., Struble G.T., Springer B.A., Emanuel S.L., Pandey N., Gruninger R.H., Adams M. (2008). 4-Amino-6-arylamino-pyrimidine-5-carbaldehyde hydrazones as potent ErbB-2/EGFR dual kinase inhibitors. Bioorg Med. Chem. Lett..

[49] Iqbal N., Iqbal N. (2014). Imatinib: A breakthrough of targeted therapy in cancer. Chemother. Res. Pract..

[50] Takimoto C.H., Awada A. (2008). Safety and anti-tumor activity of sorafenib (Nexavar) in combination with other anti-cancer agents: A review of clinical trials. Cancer Chemother. Pharmacol..

[51] Barker A.J., Gibson K.H., Grundy W., Godfrey A.A., Barlow J.J., Healy M.P., Woodburn J.R., Ashton S.E., Curry B.J., Scarlett L. (2001). Studies leading to the identification of ZD1839 (Iressa™): An orally active, selective epidermal growth factor receptor tyrosine kinase inhibitor targeted to the treatment of cancer. Bioorg Med. Chem. Lett..

[52] D’Arcangelo M., Cappuzzo F. (2013). Erlotinib in the first-line treatment of non-small-cell lung cancer. Expert. Rev. Anticancer. Ther..

[53] Ghosh N., Chaki R., Mandal V., Mandal S.C. (2010). COX-2 as a target for cancer chemotherapy. Pharmacol. Rep..

[54] Vosooghi M., Amini M. (2014). The discovery and development of cyclooxygenase-2 inhibitors as potential anticancer therapies. Expert. Opin. Drug Discov..

[55] Akl M.M., Ahmed A. (2024). Cytobiological Alterations Induced by Celecoxib as an Anticancer Agent for Breast and Metastatic Breast Cancer. Adv. Pharm. Bull..

[56] Elder D.J., Halton D.E., Hague A., Paraskeva C. (1997). Induction of apoptotic cell death in human colorectal carcinoma cell lines by a cyclooxygenase-2 (COX-2)-selective nonsteroidal anti-inflammatory drug: Independence from COX-2 protein expression. Clin. Cancer Res..

[57] Rollas S., Küçükgüzel S.G. (2007). Biological activities of hydrazone derivatives. Molecules.

[58] Liang Z., Zhang D., Ai J., Chen L., Wang H., Kong X., Zheng M., Liu H., Luo C., Geng M. (2011). Identification and synthesis of N’-(2-oxoindolin-3-ylidene)hydrazide derivatives against c-Met kinase. Bioorg Med. Chem. Lett..

[59] George R.F. (2018). Facile synthesis of simple 2-oxindole-based compounds with promising antiproliferative activity. Fut. Med. Chem..

[60] Peterson Q.P., Hsu D.C., Goode D.R., Novotny C.J., Totten R.K., Hergenrother P.J. (2009). Procaspase-3 activation as an anti-cancer strategy: Structure_activity relationship of procaspase-activating compound 1 (PAC-1) and Its cellular co-localization with caspase-3. J. Med. Chem..

[61] Abdel-Aziz A.A.-M., El-Azab A.S., AlSaif N.A., Obaidullah A.J., Al-Obaid A.M., Al-Suwaidan I.A. (2021). Synthesis, potential antitumor activity, cell cycle analysis, and multitarget mechanisms of novel hydrazones incorporating a 4-methylsulfonylbenzene scaffold: A molecular docking study. J. Enzyme Inhib. Med. Chem..

[62] Şenkardeş S., Han M.İ., Kulabaş N., Abbak M., Çevik Ö., Küçükgüzel İ., Küçükgüzel Ş.G. (2020). Synthesis, molecular docking and evaluation of novel sulfonyl hydrazones as anticancer agents and COX-2 inhibitors. Mol. Divers..

[63] Abdel-Aziz A.A.-M., El-Azab A.S., Abu El-Enin M.A., Almehizia A.A., Supuran C.T., Nocentini A. (2018). Synthesis of novel isoindoline-1,3-dione-based oximes and benzenesulfonamide hydrazones as selective inhibitors of the tumor-associated carbonic anhydrase IX. Bioorg Chem..

[64] El-Azab A.S., Abdel-Aziz A.A.-M., Bua S., Nocentini A., Bakheit A.H., Alkahtani H.M., Hefnawy M.M., Supuran C.T. (2023). Design, synthesis, and carbonic anhydrase inhibition activities of Schiff bases incorporating benzenesulfonamide scaffold: Molecular docking application. Saudi Pharm. J..

[65] El-Azab A.S., Abdel-Aziz A.A.-M., Ghabbour H.A., Bua S., Nocentini A., Alkahtani H.M., Alsaif N.A., Al-Agamy M.H.M., Supuran C.T. (2022). Carbonic Anhydrase Inhibition Activities of Schiff's Bases Based on Quinazoline-Linked Benzenesulfonamide. Molecules.

[66] El-Azab A.S., Abdel-Aziz A.A.-M., Bua S., Nocentini A., Alanazi M.M., AlSaif N.A., Al-Suwaidan I.A., Hefnawy M.M., Supuran C.T. (2019). Synthesis and comparative carbonic anhydrase inhibition of new Schiff's bases incorporating benzenesulfonamide, methanesulfonamide, and methylsulfonylbenzene scaffolds. Bioorg Chem..

[67] El-Azab A.S., Abdel-Aziz A.A.-M., Bua S., Nocentini A., El-Gendy M.A., Mohamed M.A., Shawer T.Z., AlSaif N.A., Supuran C.T. (2019). Synthesis of benzensulfonamides linked to quinazoline scaffolds as novel carbonic anhydrase inhibitors. Bioorg Chem..

[68] Shoemaker R.H. (2006). The NCI60 human tumour cell line anticancer drug screen. Nature reviews. Nat. Rev. Cancer.

[69] Abdel-Aziz A.A.-M., El-Azab A.S., Abou-Zeid L.A., ElTahir K.E., Abdel-Aziz N.I., Ayyad R.R., Al-Obaid A.M. (2016). Synthesis, anti-inflammatory, analgesic and COX-1/2 inhibition activities of anilides based on 5,5-diphenylimidazolidine-2,4-dione scaffold: Molecular docking studies. Eur. J. Med. Chem..

[70] Pfeffer C.M., Singh A.T.K. (2018). Apoptosis: A target for anticancer therapy. Int. J. Mol. Sci..

[71] Vermes I., Haanen C., Steffens-Nakken H., Reutelingsperger C. (1995). A novel assay for apoptosis flow cytometric detection of phosphatidylserine expression on early apoptotic cells using fluorescein labelled annexin V. J. Immunol. Methods.

[72] Kim K.H., Sederstrom J.M. (2015). Assaying cell cycle status using flow cytometry. Curr. Protoc. Mol. Biol..

[73] El-Azab A.S., Mary Y.S., Panicker C.Y., Abdel-Aziz A.A.-M., El-Sherbeny M.A., Van Alsenoy C. (2016). DFT and experimental (FT-IR and FT-Raman) investigation of vibrational spectroscopy and molecular docking studies of 2-(4-oxo-3-phenethyl-3, 4-dihydroquinazolin-2-ylthio)-N-(3, 4, 5-trimethoxyphenyl) acetamide. J. Mol. Struct..

[74] William L.J., David S.M., Julian T.-R. (1996). Development and Testing of the OPLS All-Atom Force Field on Conformational Energetics and Properties of Organic Liquids. J. Am. Chem. Soc..

[75] Brogi S., Ramunno A., Savi L., Chemi G., Alfano G., Pecorelli A., Pambianchi E., Galatello P., Compagnoni G., Focher F. (2017). First dual AK/GSK-3β inhibitors endowed with antioxidant properties as multifunctional, potential neuroprotective agents. Eur. J. Med. Chem..

[76] Vallone A., D’Alessandro S., Brogi S., Brindisi M., Chemi G., Alfano G., Lamponi S., Lee S.G., Jez J.M., Koolen K.J.M. (2018). Antimalarial agents against both sexual and asexual parasites stages: Structure-activity relationships and biological studies of the Malaria Box compound 1-[5-(4-bromo-2-chlorophenyl)furan-2-yl]-N-[(piperidin-4-yl)methyl]methanamine (MMV019918) and analogues. Eur. J. Med. Chem..

[77] da Silva E.R., Brogi S., Lucon-Júnior J.F., Campiani G., Gemma S., Maquiaveli C.D.C. (2019). Dietary polyphenols rutin, taxifolin and quercetin related compounds target Leishmania amazonensis arginase. Food Funct..

[78] Friesner R.A., Banks J.L., Murphy R.B., Halgren T.A., Klicic J.J., Mainz D.T., Repasky M.P., Knoll E.H., Shelley M., Perry J.K. (2004). Glide: A new approach for rapid, accurate docking and scoring. 1. Method and assessment of docking accuracy. J. Med. Chem..

[79] Brogi S., Fiorillo A., Chemi G., Butini S., Lalle M., Ilari A., Gemma S., Campiani G. (2017). Structural characterization of Giardia duodenalis thioredoxin reductase (gTrxR) and computational analysis of its interaction with NBDHEX. Eur. J. Med. Chem..

[80] William L.J., Jayaraman C., Jeffry D.M., Roger W.I., Michael L.K. (1983). Comparison of simple potential functions for simulating liquid water. J. Chem. Phys..

[81] Sirous H., Chemi G., Gemma S., Butini S., Debyser Z., Christ F., Saghaie L., Brogi S., Fassihi A., Campiani G. (2019). Identification of Novel 3-Hydroxy-pyran-4-One Derivatives as Potent HIV-1 Integrase Inhibitors Using in silico Structure-Based Combinatorial Library Design Approach. Front. Chem..

[82] Darryl D.H., Richard A.F., Bruce J.B. (1994). A Multiple-Time-Step Molecular Dynamics Algorithm for Macromolecules. J. Phys. Chem..

[83] Hoover W.G. (1985). Canonical dynamics: Equilibrium phase-space distributions. Phys. Rev. A Gen. Phys..

[84] Glenn J.M., Douglas J.T., Michael L.K. (1994). Constant pressure molecular dynamics algorithms. J. Chem. Phys..

[85] Ulrich E., Lalith P., Max L.B., Tom D., Hsing L., Lee G.P. (1995). A smooth particle mesh Ewald method. J. Chem. Phys..

[86] Grever M.R., Schepartz S.A., Chabner B.A. (1992). The National Cancer Institute: Cancer drug discovery and development program. Semin. Oncol..

[87] Abdel-Rahman I.M., Mustafa M., Mohamed S.A., Yahia R., Abdel-Aziz M., Abuo-Rahma G.E.A., Hayallah A.M. (2021). Novel Mannich bases of ciprofloxacin with improved physicochemical properties, antibacterial, anticancer activities and caspase-3 mediated apoptosis. Bioorg. Chem..

[88] El-Sayed M.A., Abdel-Aziz N.I., Abdel-Aziz A.A., El-Azab A.S., Asiri Y.A., Eltahir K.E. (2011). Design, synthesis, and biological evaluation of substituted hydrazone and pyrazole derivatives as selective COX-2 inhibitors: Molecular docking study. Bioorganic Med. Chem..

[89] Uddin M.J., Rao P.N., Knaus E.E. (2004). Design and synthesis of acyclic triaryl (Z)-olefins: A novel class of cyclooxygenase-2 (COX-2) inhibitors. Bioorganic Med. Chem..

[90] Manning G., Whyte D.B., Martinez R., Hunter T., Sudarsanam S. (2002). The protein kinase complement of the human genome. Science.

[91] Grant S.K. (2009). Therapeutic protein kinase inhibitors. Cell. Mol. Life Sci. CMLS.

[92] Nakamura J.L. (2007). The epidermal growth factor receptor in malignant gliomas: Pathogenesis and therapeutic implications. Expert Opin. Ther. Targets.

[93] Wang J., Lenardo M.J. (2000). Roles of caspases in apoptosis, development, and cytokine maturation revealed by homozygous gene deficiencies. J. Cell Sci..

[94] Mitupatum T., Aree K., Kittisenachai S., Roytrakul S., Puthong S., Kangsadalampai S., Rojpibulstit P. (2016). mRNA Expression of Bax, Bcl-2, p53, Cathepsin B, Caspase-3 and Caspase-9 in the HepG2 Cell Line Following Induction by a Novel Monoclonal Ab Hep88 mAb: Cross-Talk for Paraptosis and Apoptosis. Asian Pac. J. Cancer Prev..

[95] Elshemy H.A.H., Zaki M.A. (2017). Design and synthesis of new coumarin hybrids and insight into their mode of antiproliferative action. Bioorg. Med. Chem..

[96] Mohamed H., Heba A.H., Hesham A.M.G., Bahaa G.M.Y., Alaa M.H., Mohamed A.-A. (2022). Structure-based design, synthesis and antiproliferative action of new quinazoline-4-one/chalcone hybrids as EGFR inhibitors. J. Mol. Struct..

[97] Abou-Zied H.A., Youssif B.G.M., Mohamed M.F.A., Hayallah A.M., Abdel-Aziz M. (2019). EGFR inhibitors and apoptotic inducers: Design, synthesis, anticancer activity and docking studies of novel xanthine derivatives carrying chalcone moiety as hybrid molecules. Bioorg Chem..

