# Supplementary material for: Antitumor Activity and Multi-Target Mechanism of Phenolic Schiff Bases Bearing Methanesulfonamide Fragments: Cell Cycle Analysis and a Molecular Modeling Study
Source: Int J Mol Sci. 2024 Dec 19;25(24):13621. doi: 10.3390/ijms252413621 (PMC11728000; doi:10.3390/ijms252413621)
Supplement: Supplementary file 1 [file ijms-25-13621-s001.zip › ijms-3339929-supplementary.pdf]

Antitumor Activity and Multi-Target Mechanism of Phenolic Schiff Bases Bearing Methanesulfonamide Fragments: Cell Cycle Analysis and a Molecular Modeling Study

Alaa A.-M. Abdel-Aziz*, Adel S. El-Azab, Simone Brogi, Rezk R. Ayyad,

Ibrahim A. Al-Suwaidan, Mohamed Hefnawy

Biological experiments

1. *In vitro* NCI anticancer screening [86,68]

The methodology of the NCI anticancer screening has been described in detail elsewhere (https://dtp.cancer.gov/discovery_development/nci-60/methodology.htm). Briefly, the primary anticancer assay was performed at approximately 60 human tumor cell line panels derived from nine neoplastic diseases following the protocol of the Drug Evaluation Branch, National Cancer Institute, Bethesda. Tested compounds were added to the culture at a single concentration (10^{-5} M), and the cultures were incubated for 48 h. End determinations were made with a protein-binding dye, SRB. Results for each tested compound were reported as the percent of growth of the treated cells compared to the untreated control cells. The percentage growth was evaluated spectrophotometrically versus controls not treated with test agents. The cytotoxic and/or growth inhibitory effects of the most active selected compound were tested *in vitro* against the full panel of about 60 human tumor cell lines at 10-fold dilutions of five concentrations ranging from 10^{-4} to 10^{-8} M. A 48-h continuous drug exposure protocol was followed. An SRB protein assay was used to estimate cell viability or growth. Using the seven absorbance measurements [time zero (Tz), control growth in the absence of drug (C), and test growth in the presence of drug at the five concentration levels (Ti)], the percentage growth was calculated at each of the drug concentration levels. Percentage growth inhibition was calculated as: $[(Ti - Tz)/(C - Tz)] - 100$ for concentrations for which Ti > Tz, and $[(Ti - Tz)/Tz] - 100$ for concentrations for which Ti < Tz. Three-dose response parameters were calculated for each compound. Growth inhibition of 50% (GI_{50}) was calculated from $[(Ti - Tz)/(C - Tz)] - 100 = 50$, which is the drug concentration resulting in a 50% lower net protein increase in the treated cells (measured by SRB staining) as compared to the net protein increase seen in the control cells. The drug concentration resulting in TGI was calculated from Ti = Tz. The LC_{50} (concentration of drug resulting in a 50% reduction in

the measured protein at the end of the drug treatment as compared to that at the beginning) indicating a net loss of cells following treatment was calculated from $[(Ti - Tz)/Tz] - 100 = - 50$. Values were calculated for each of these three parameters if the activity level was reached; however, if the effect was not reached or exceeded, that parameter's value was expressed as more or less than the maximum or minimum concentration tested. The log GI_{50}, log TGI, and log LC_{50} were then determined and defined as the mean of the logs of the individual GI_{50}, TGI, and LC_{50} values. The lowest values are obtained with the most sensitive cell lines. The compound with log GI_{50} values −4 and <−4 was declared active.

2. *In vitro* cytotoxic activity towards normal fibroblast (WI-38) cells [87]

Cell Lines were obtained from the American Type Culture Collection; cells were cultured using DMEM (Invitrogen/Life Technologies) supplemented with 10% FBS (Hyclone), ten ug/ml of insulin (Sigma), and 1% penicillin–streptomycin. Cultures were removed from the incubator into a laminar flow hood, reconstituted with 3 mL of balanced salt solution without phenol red and serum, and then added to an amount equal to 10% of the culture medium volume. Cultures returned to the incubator for 2–4 h. After the incubation period, the resulting formazan crystals dissolved by adding an amount of MTT Solubilization Solution [M-8910] equal to the original culture medium volume. The absorbance was measured Spectrophotometrically at a wavelength of 570 nm.

3. *In vitro* cyclooxygenase (COX-2) inhibition assay [88,89]

The ability of the test compounds to inhibit COX-2 was determined using an enzyme immunoassay (EIA) (kit catalog number 560101, Cayman Chemical, Ann Arbor, MI, USA) according to the manufacturer’s instructions.

Cyclooxygenase catalyzes the first step in the biosynthesis of arachidonic acid (AA) to PGH_2. $PGF_{2\alpha}$, produced from PGH_2 by reduction with stannous chloride, is measured by enzyme immunoassay (ACE™ competitive EIA). Stock solutions of test compounds were dissolved in a minimum volume of DMSO. Briefly, a series of supplied reaction buffer solutions (960 µL, 0.1 M Tris–HCl pH 8.0 containing 5 mM EDTA and 2 mM phenol) with COX-2 (10 µL) enzyme in the presence of heme (10 µL) were added 10 µL of various concentrations of test drug solutions (0.01, 0.1, 1, 10, 50, and 100 µM in a final volume of 1 mL). These solutions were incubated for 5 min at 37 °C after which 10 µL of AA (100 µM) solution was added, and the COX-2 reaction was stopped by the addition of 50 µL of 1 M HCl after 2 min. $PGF_{2\alpha}$, produced from PGH_2 by reduction with stannous chloride, was measured by enzyme immunoassay. This assay is based on the competition between PGs and a PG-acetylcholinesterase conjugate (PG tracer) for a limited amount of PG antiserum. The amount of PG tracer, able to bind to the PG antiserum, is inversely proportional to the concentration of PGs in the wells since the concentration of PG tracer is held constant while the concentration of PGs varies. This antibody–PG complex binds to a mouse anti-rabbit monoclonal antibody previously attached to the well. The plate is washed to remove any unbound reagents, and then Ellman's reagent, which contains the substrate of acetylcholine esterase, is added to the well. The product of this enzymatic reaction produces a distinct yellow color that absorbs at 410 nm. The intensity of this color, determined spectrophotometrically, is proportional to the amount of PG tracer bound to the well, which is inversely proportional to the number of PGs present in the well during the incubation: Absorbance α [Bound PG Tracer] α1/PGs. Percent inhibition was calculated by comparing the compound treated to various control incubations. The concentration of the test compound causing 50% inhibition (IC_{50}, µM) was calculated from the concentration–inhibition response curve (duplicate determinations).

4. *In vitro* EGFR/HER2 kinase inhibition assay [47,90–92]

In vitro luminescent EGFR tyrosine kinase assay using Kinase-Glo® M.A.X. as a detection reagent and *In vitro* HER2 tyrosine kinase assay using DP-Glo™ reagent that measures A.D.P. formed from a kinase reaction, this luminescent signal positively correlates with A.D.P. amount and kinase activity. Briefly, Mix 6 µl Kinase assay buffer, 1 µl ATP, and 1 µl PTK substrate in 17 µl distilled water (master mixture). In Every well, mix 20 µl of the master mixture and 5 µl of Inhibitor solution (Test Inhibitor) for positive control and 5 µl of the same solution without inhibitor (Inhibitor buffer) as a blank solution, add 20 µl of diluted EGFR or Her2 enzyme and incubate at 30°C for 40 minutes. Add 50 µl of Kinase-Glo Max reagent to each well, cover the plate with aluminum foil, incubate the plate at room temperature for 15 minutes, and measure luminescence using the microplate reader. "Blank" value is subtracted from all readings [71,72,93]. All samples and controls should be tested in duplicate.

5. Annexin V–FITC apoptosis assay [47,71]

The apoptosis induction was evaluated using the well-established method of the Annexin 5-FITC/PI apoptosis detection kit Bio Vision Research, Mountain View, CA 94,043 USA, according to the manufacturer's instructions. Breast carcinoma (MCF-7) cell were used for this assay, and the FACSCalibur flow cytometer was used for analysis [95,96]. Briefly, 4×10^6 cell/T 75 flasks were exposed to compound **8** at its IC_{50} concentration for 24 h. The cells were then collected by trypsinization, and 0.5×10^6 cells were washed twice with PBS and stained with 5 µL Annexin V-FITC and 5 µL PI in 1 × binding buffer for 15 min at room temperature in the dark. Analysis was performed using a FACS Calibur flow cytometer. Data were collected using logarithmic amplification of both the FL1 (FITC) and the FL2 (PI) channels. Quadrant analysis of coordinate dot plots was performed with CellQuest software. Unstained cells were used to adjust the

photomultiplier voltage and for compensation setting adjustment to eliminate spectral overlap between the FL1 and the FL2 signals.

6. Cell cycle analysis [47,72]

Breast carcinoma (MCF-7) cell was seeded at 2 × 105 cells/well density and incubated for 24 h in six-well plates. Foetal bovine serum (10%) was added, and the cells were incubated at 37 °C in an atmosphere of 5% CO2. The medium was replaced with 1% (v/v) DMSO containing 10.0 µM of compound **8**; the cells were then incubated for 48 h, washed with phosphate-buffered saline, fixed with 70% ethanol, rinsed with phosphate-buffered saline, and then stained with the DNA fluorochrome propidium iodide (PI) for 15 min at 37 °C. Then, samples were analyzed by flow cytometry using a FACSCalibur (Becton Dickinson) [96,97]. The cell cycle distributions were calculated using CellQuest software (Becton Dickinson).

7. Caspase-8 and 9 activation assay [93,94]

A breast carcinoma (MCF-7) cell line was obtained from ATCC. RPMI 1640 containing 10% FBS allowed cells to grow at 37 °C, stimulated with the compounds to be tested for caspase-8 or caspase-9 and lysed with Cell Extraction Buffer. Standard Diluent Buffer was used to dilute the lysate over the range of the assay and measure human active caspase-8 or caspase-9 content. (cells are Plated in a density of 1.2-1.8 X 10,000 cells/well in a volume of 100 lL complete growth medium + 100 lL of the tested compound per well in a 96-well plate for 24/48 h before the enzyme assay).

8. Evaluation of Bax and Bcl-2 expressions [95–97]

m RNA isolation was carried out using an RNeasy extraction kit, up to 1 X 10^7 cells. They were disrupted in Buffer RLT and homogenized. Ethanol was added to the lysate to promote the selective binding of RNA to the RNeasy membrane. Then, the sample was applied to the RNeasy

Mini spin column. Total RNA bound to the membrane. High-quality RNA was eluted using RNase-free water. A micro-centrifuge was used to centrifuge all binds, wash, and elution steps. Kit contents were BIORAD iScriptTM One-Step Real-Time RT-PCR Kit with SYBR$^{@}$ Green. The reagent used is described as follows: iScript Reverse Transcriptase Optimized 50X formulation of iScript MMLV for One-Step RT-PCR reverse transcriptase for One-Step RTPCR procedures (yellow cap) 2X SYBR_ Green RT-PCR 2X reaction buffer containing 0.4 mM of each dNTP (dATP, Reaction Mix dCTP, dGTP, dTTP), magnesium chloride, iTaq DNA (green cap) polymerase, 20 nM fluorescein, SYBR_ Green I dye, stabilizers Nuclease-free H_2O. A reaction mix (50 lL) was prepared according to the following recipe: 2X Sybr Green RT-PCR Master (25 lL), (10 lM) forward primer (1.5 10 lL), (10 lM) Reverse primer (1.5 lL), Nuclease-free H2O (11 lL), RNA template (1 pg to 100 ng total RNA) (10 lL) and iScript Reverse Transcriptase for One-Step RT-PCR (1 lL). Amplification was performed using 7500 Fast RT-PCR Systems (Applied Biosystems, USA) 10 ng of cDNA using a Power Sybr Green PCR Master MIX (Applied Biosystems). The amplification protocol was as follows: cDNA synthesis: 50 ºC (10 min), iScript Reverse transcriptase inactivation: 95 ºC (5 min), PCR cycling and detection (40 cycles): 95 ºC (10 s), data collection step: 60 ºC (30 s), melt curve analysis: 95 _C (1 min), 55 ºC (1 min) and 55 ºC (10 s) (80 cycles, increasing each by 0.5 °C each cycle). Then, the products were routinely checked using dissociation curve software. Transcript quantities were compared using the relative Ct method, and the amount of BAX and BCL2 were normalized to the endogenous control (GAPDH). The value concerning the control sample was given, and real-time PCR primer sequences were as follows:

This report was generated by Rotor-Gene 6000 Series Software 1.7 (Build 87)
Copyright ©2000-2006 Corbett Research, a Division of Corbett Life Science. All rights reserved.
ISO 9001:2000 (Reg. No. QEC21313)

Primers

<u>primers</u>

[redacted]

Bax : F 5’- TCAGGATGCGTCCACCAAGAAG -3’,

Bax : R 5'- TGTGTCCACGGCGGCAATCATC-3'.

[redacted]

Bcl2 : F 5’- ATCGCCCTGTGGATGACTGAGT -3’,

Bcl2 : R 5'- GCCAGGAGAAATCAAACAGAGGC -3'.

[redacted]

Casp8 : F 5’- AGAAGAGGGTCATCCTGGGAGA -3’,

Casp8 : R 5'- TCAGGACTTCCTTCAAGGCTGC -3'.

[redacted]

Casp9 : F 5’- GTTTGAGGACCTTCGACCAGCT -3’,

Casp9 : R 5'- CAACGTACCAGGAGCCACTCTT-3'.

[redacted]

GAPDH : F 5’- GTCTCCTCTGACTTCAACAGCG-3’

GAPDH : R 5’- ACCACCCTGTTGCTGTAGCCAA-3’

9. Statistical analysis

Prism 5 program was used to statistically analyze data using a one-way ANOVA test followed by Tukey's as post-ANOVA for multiple comparisons at $P \leq .05$. Data were presented as mean ± SEM.
